# Long-Range Transport of Oil by Marine Plastic Debris: Evidence from an 8500 km Journey

**DOI:** 10.1021/acs.est.5c14571

**Published:** 2026-01-07

**Authors:** Bryan D. James, Luis E. A. Bezerra, Diane Buhler, Rivelino M. Cavalcante, Martha L. Aguilera, Bing Chen, Jonas Gros, Ulrich M. Hanke, Karin L. Lemkau, Robert K. Nelson, Sydney F. Niles, André H. B. de Oliveira, Thomas D. Pitchford, Jagoš R. Radović, Ryan P. Rodgers, Marcelo O. Soares, Scott A. Socolofsky, Roger E. Summons, Robert F. Swarthout, Carlos E. P. Teixeira, David L. Valentine, Helen K. White, Min Yang, Eliete Zanardi-Lamardo, Baiyu Zhang, Christopher M. Reddy

**Affiliations:** † Department of Chemical Engineering, 1848 Northeastern University, Boston, Massachusetts 02115, United States; ‡ Department of Marine Chemistry and Geochemistry, 10627 Woods Hole Oceanographic Institution, Woods Hole, Massachusetts 02543, United States; § Instituto de Ciências do Mar-LABOMAR, Federal University of Ceará, Fortaleza CE 60165-081, Brazil; ∥ Friends of Palm Beach, Palm Beach, Florida 33480, United States; ⊥ Ion Cyclotron Resonance Program, National High Magnetic Field Laboratory, 7823 Florida State University, Tallahassee, Florida 32310, United States; # Northern Region Persistent Organic Pollutant Control (NRPOP) Laboratory, Faculty of Engineering and Applied Science, Memorial University, St. John’s, Newfoundland and Labrador A1B3X5, Canada; ¶ Independent Researcher, Villars-sur-Glâne 1752, Switzerland; ∇ Department of Chemistry, 1632 Western Washington University, Bellingham, Washington 98225, United States; ○ Universite de Pau et des Pays de l’Adour, E2S UPPA, CNRS, IPREM, UMR 5254, 2 Avenue du Président Pierre Angot, Pau 64053, France; ⧫ Department of Analytical Chemistry and Physical Chemistry, Federal University of Ceará, Fortaleza CE 60440900, Brazil; †† Center for Petroleum Geochemistry (UH-CPG), Department of Earth and Atmospheric Sciences, 14743 University of Houston, Houston, Texas 77204, United States; ‡‡ Zachry Department of Civil and Environmental Engineering, Texas A&M University, College Station, Texas 77843, United States; §§ Department of Earth, Atmospheric, and Planetary Sciences, Massachusetts Institute of Technology, Cambridge, Massachusetts 02142, United States; ∥∥ Department of Chemistry and Fermentation Sciences, 1801 Appalachian State University, Boone, North Carolina 28608, United States; ⊥⊥ Marine Science Institute, 8786 University of California-Santa Barbara, Santa Barbara, California 93106, United States; ## Department of Earth Science, University of California-Santa Barbara, Santa Barbara, California 93106, United States; ¶¶ Department of Chemistry, 3776 Haverford College, Haverford, Pennsylvania 19041, United States; ∇∇ Department of Oceanography, Federal University of Pernambuco, Recife, PE 50670-901, Brazil

**Keywords:** oil spills, marine debris, plastic pollution, cocontaminants, citizen science, transboundary transport

## Abstract

Weathering processes typically restrict the distance spilled oil travels to a few hundred kilometers in the ocean. Leveraging oiled marine debris as “drifters of opportunity”, we tested the hypothesis of the unprecedented long-range (thousands of kilometers) transequatorial transport of oil adhered to marine debris by surface currents. Dispersion modeling backed by historical drift bottle experiments supported the plausibility for this hypothesis, and molecular forensics provided the definitive evidence proving it. Oil carried by marine debris arriving at Palm Beach, Florida in 2020 matched oil from the 2019 Brazil mystery oil spill, having traveled ∼8500 km in ∼240 days. We demonstrate an additive contaminant effect whereby plastic pollution facilitates the long-range transport of oil pollution. These findings underscore that regional inputs into the global ocean can have transboundary impacts.

## Graphical abstract

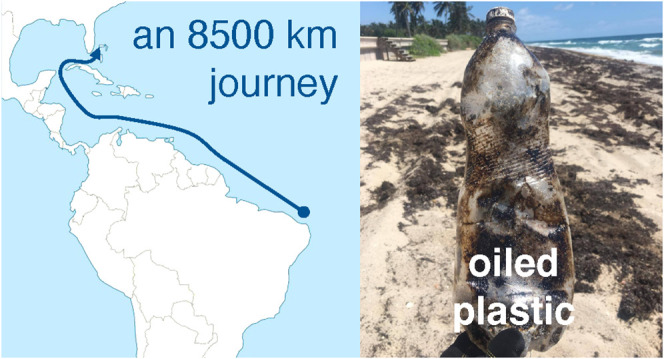

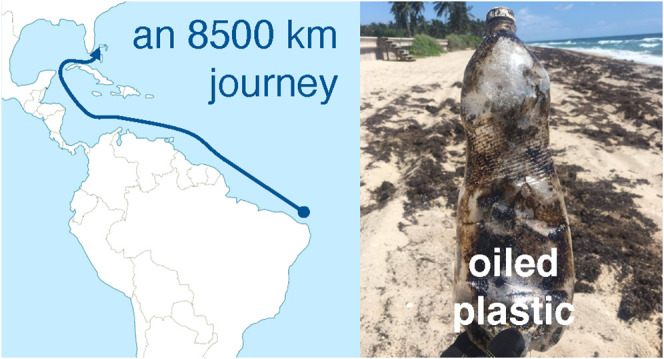

## Introduction

Palm Beach, Florida, USA, is the easternmost point of the Florida peninsula and five to seven km from the flow of the Gulf Stream centered between Florida and the Bahamas. Onshore winds deliver plastic, bitumen, *Sargassum*, and seeds to the region, making it one of the most debris-ridden beaches in the Southeast United States.^1–3^ Numerous organizations and community groups track and remove beached debris along the southeast coast of Florida.

From late May through September 2020, the Friends of Palm Beach (FOPB), a nonprofit organization dedicated to cleaning the coastline of Palm Beach, noticed the arrival of an unusually large quantity of capped glass and plastic bottles partially or fully covered with black residue (Figure 1A). The black residues on the bottles ranged from thin coatings to several millimeters thick. When present and legible, the labels on the bottles were written in Portuguese, Spanish, or English, with some identified as consumer products mainly from South America and Caribbean countries (Figure S1). During this same period, in July and August 2020, large rubber bales arrived in Palm Beach (Figure 1B), as well as Ambergris Caye, Belize, and Boca Chica, Texas, USA, receiving coverage from local media outlets (Figure 2).^4^

**Figure 1:**
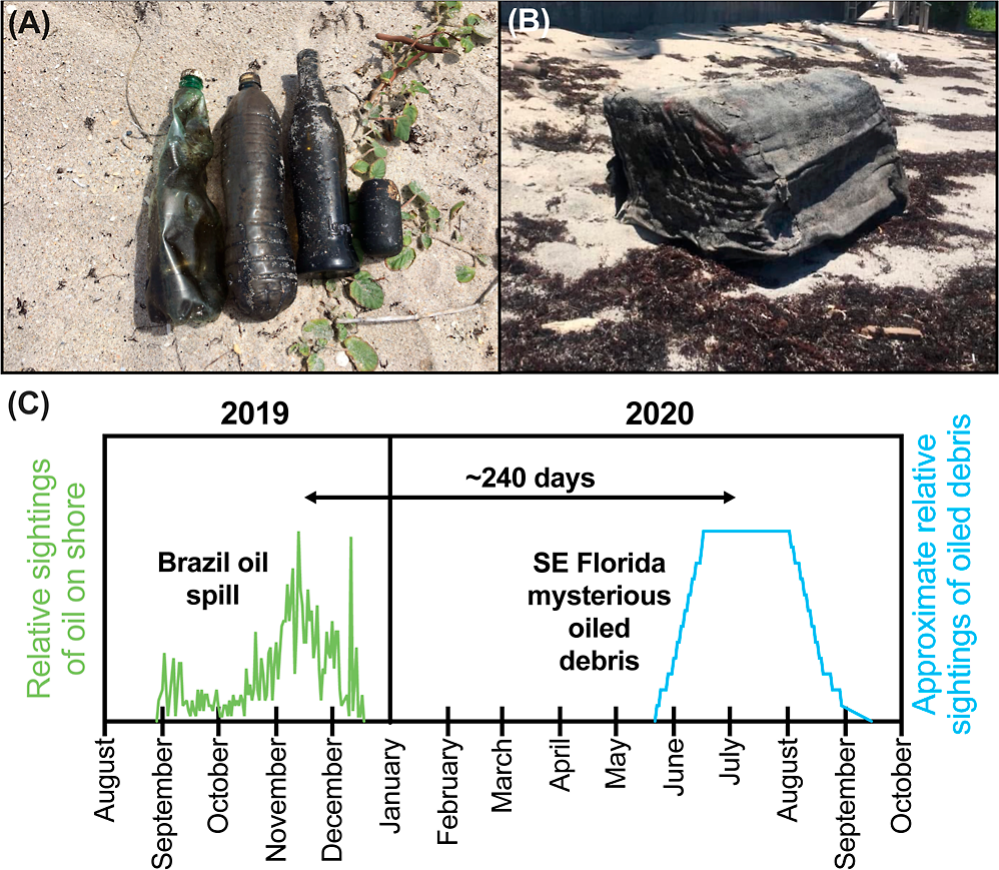
Plastic and glass bottles with black residue. (A) Photograph of representative marine debris with black residues collected by the FOPB on July 27, 2020, from beaches in Palm Beach, Florida. (B) Photograph of a rubber bale washed ashore in Palm Beach, Florida, in August 2020. (C) Timelines of the relative extent of oiling of new beaches during the Brazil oil spill^5^ and the approximate relative extent of marine debris with black residues collected by FOPB.

**Figure 2:**
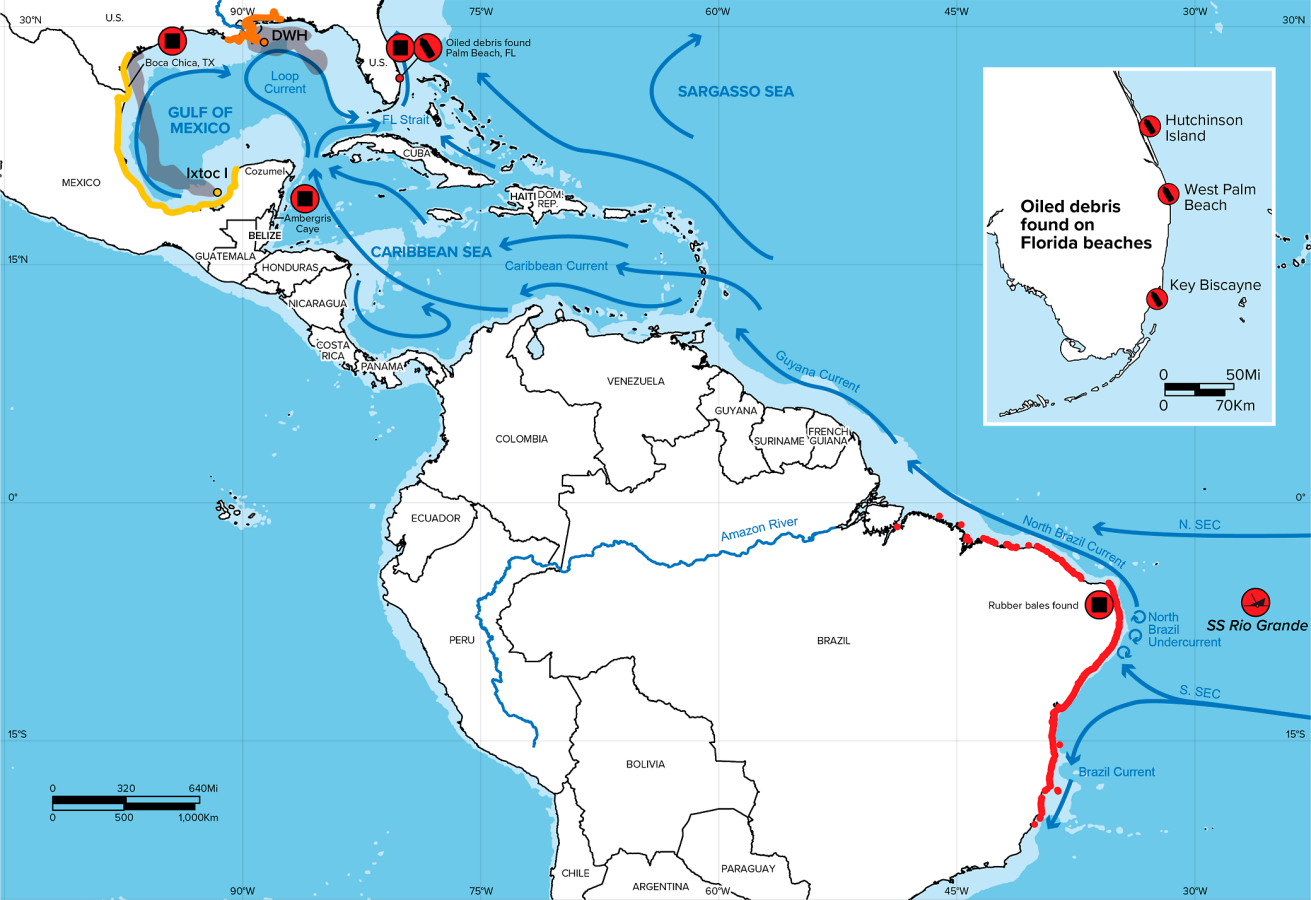
Map of the sightings of the oiled debris and rubber bales. Oiled bottles and other oiled debris were found in Hutchinson Island, FL, West Palm Beach, FL, and Key Biscayne, FL (inset; red circles with black bottle icon). Rubber bales were found in Palm Beach, FL, Boca Chica, TX, Ambergris Caye, Belize, and along the North and Northeast Brazilian coast (red circles with black square icons). Three notable oil spills and their extent of coastline oiling are highlighted as well: the 2019 Brazil oil spill (in red),^5^ the 2010 Deepwater Horizon (DWH) oil spill (in orange),^20,21^ and the 1979 *Ixtoc I* oil spill (in yellow).^20,21^

At the peak of their stranding, 10 to 12 black residue-coated items were removed per daily cleanup in June and July, and this number tapered to one to three items by the middle of September 2020 (Figure 1C). While the FOPB occasionally observed black-stained debris and tar-covered pieces, this event was unprecedented due to the sustained daily arrival of various pieces of debris with black residue over several months. There was no known local source when government officials were contacted, and no oil spills were known locally within the investigated period. Due to the unusual appearance, the FOPB posted updates and images of the debris on social media, calling it “TarTrash” with the hashtags #COVEREDINTRASH, #TARCOVERED, and #TOXICTRASH.^6–8^ Responding to the post by the FOPB, other groups monitoring ∼200 km of beachfront in Southeast Florida noticed similar debris (Figure 2, inset).^9^

The 2020 Palm Beach mystery debris may be linked to two other mysterious pollution events from one to two years earlier along the Brazilian coastline. Beginning in October 2018, rubber bales with sizes from 0.4 × 0.4 × 0.4 m to 1.5 × 1.5 × 1.5 m and weighing up to 200 kg appeared along Brazil’s north and northeast coastline (Figure 2).^10^ The following year, from August to December 2019, over 3000 km of Brazil’s coastline was impacted by oil (Figure 2), thus becoming the worst oil spill in the country’s history.^11–15^ Some of the earliest reported locations for oiling were where the bales had been found the previous year. Brazilian authorities suspected four crude oil tankers as potential culprits.^11,12^ Members of our team hypothesized that the oil and bales came from the SS *Rio Grande*, a German supply ship sunk by the U.S. Navy in January 1944, residing 1000 km off the Brazilian coastline at a depth of 5762 m (Figure 2).^10^ The SS *Rio Grande* is one of the thousands of World War II naval shipwrecks that have been called “ticking ecological time bombs” because of their potential to release the oil remaining in their tanks.^16,17^ Still, the source of the oil remains a mystery. The simultaneous arrival of oil and rubber bales in Palm Beach during the summer of 2020 raised the question of whether the oil on the debris originated from the 2019 Brazil oil spill.

In the event of an oil spill, the oil rarely travels more than 300 km from the source due to its removal by natural processes (weathering) and/or engineered spill response measures (e.g., dispersant application).^18,19^ In this context, we tested the hypothesis of unprecedented long-range (∼8500 km) transequatorial transport of oil adhered to floating marine debris by surface currents.

## Materials and Methods

### Numerical Modeling of Possible Trajectories

Trajectories of virtual particles were simulated using the OpenDrift model to evaluate the paths and potential source regions for the oiled debris. The OpenDrift dispersion model is a software package for modeling the trajectories and fate of objects or substances (e.g., oil and its weathering, microplastics, larvae) drifting in the ocean.^22^ OpenDrift uses a Runge–Kutta fourth-order time-stepping method to calculate particle positions based on input ocean circulation data.

For the dispersion simulations, we used daily mean currents 3D data from the GLORYS12 V1 Global Ocean Physics Reanalysis produced by the Mercator-Ocean system using a 1/12° spatial resolution (∼8 km) and 50 vertical levels ranging from 0 to 5500 m (hereafter referred to as Mercator).^23^ The GLORYS12 V1 vertical velocities were not used and vertical mixing was included in the simulation using the KPP Scheme. The Mercator Model is widely used and validated for the Atlantic Ocean.^10,24–26^ Wind data from the Global Ocean Hourly Reprocessed Sea Surface Wind and Stress from Scatterometer and Model^27^ was used to include windage in the simulation with a 2% wind drift as in previous studies.^28–30^ Waves were not included in the simulations.

Using the OceanDrift model, we simulated the trajectories of 10,000 neutrally buoyant, virtual particles (i.e., oiled bottles) evenly released from August, 13, 2020 to May 15, 2020 at 50 cm from the surface on a 5 km radius around latitude 26.7 °N, and longitude 80 °W. A time step of 60 min was used with no additional diffusion added to the trajectories. The particles were followed backward in time for one year. Particles were considered stranded when they reached a coastline.

### Analytical Dispersion Modeling

The Okubo diagram^31^ (as published in ref 32) relates the apparent turbulent diffusion coefficient to the size of a dispersed cloud of tracer in oceanic settings. The apparent diffusion coefficient increases with the size of the tracer cloud because larger eddies become increasingly responsible for turbulent mixing rather than just chaotic advection. The Richardson model of turbulent diffusion equates the variance σ of the tracer distribution to the travel time *t* by, $\sigma^{2}=\frac{2}{3}\left(c\varepsilon \right)t^{3}$ , where (*c*ε) can be related to the slope α of the data in Okubo’s diagram by the expression found in ref 32, $\left(c\varepsilon \right)=\frac{4}{9}\alpha^{3}$ .

Depending on the data set, α varies from 0.01 cm^2/3^ s^–1^ to 0.002 cm^2/3^ s^–1^, with smaller values of α corresponding to experiments with larger dispersed clouds of tracer (longer turbulent mixing times). A tracer cloud of floating, oiled plastics would have a horizontal length scale of about *L* = ± 2σ, and will pass the Florida Straits over a period of *t* = *uL* ^–1^, where *u* is the mean current speed. Taking an average current speed of 14 km d^–1^, using the lower value of α = 0.002 cm^2/3^ s^–1^, and taking the observed contamination period of 130 d (May to August 2020; Figure 1C), we computed a total travel time from the release to the Florida event of 240 d, which agrees with the time between the Brazil oil spill and the oiled debris washing up on southeastern Florida beaches (Figure 1C).

### Samples

Samples of oiled debris that arrived in the summer of 2020 on beaches in Palm Beach, Florida, were set aside by the FOPB in the shade and shipped several weeks later to the Woods Hole Oceanographic Institution (Woods Hole, MA). Ten items were collected, which included three glass bottles (FOPB-01, -08, -09), three plastic bottles (FOPB-04, -05, -10), a piece of rubber debris (FOPB-02), a piece of plastic debris (FOPB-03), a piece of plastic film debris (FOPB-06), and a cylindrical piece of plastic debris (FOPB-07) (Figure S2; Table S1). Oily residues were scraped from three glass bottles and one plastic bottle (Figure S2), a piece of black rubber (Figure S2), and a piece of plastic debris, labeled FOPB-01, -02, -03, -05, -08, and -09, respectively (Figure S2; Table S1). The analyses performed on each sample are detailed in Table S2, which includes chemical characterization by one-dimensional gas chromatography (GC) with flame ionization detection (FID) and mass spectrometry (MS), comprehensive high-resolution two-dimensional gas chromatography (GC × GC) with FID and high-resolution time-of-flight mass spectrometry (HRT), and Fourier transform ion cyclotron resonance mass spectrometry (FT-ICR MS) as well as simulation of oil weathering.^13^

Two samples from the 2019 Brazil oil spill previously characterized were used as representative oil samples from the spill,^13^ specifically, samples Brazil-12 and Brazil-12/13. These samples represented the northernmost extent of sampling within the study and others, and were nearly devoid of sand. Due to their similar characteristics and sample size requirements, a 1:1 combination of samples 12 and 13 (Brazil-12/13) was used for some analyses in this previous study.^13^ Collectively, these samples underwent the same analytical treatment as the oily residues scraped from the bottles collected in Palm Beach, Florida, allowing for direct comparisons of the oils (Table S2). The results for these samples from the previous study^13^ were re-evaluated and replotted for comparison to the FOPB samples.

### Comprehensive Two-Dimensional Gas Chromatography (GC × GC)

Samples were analyzed by GC × GC-FID and GC × GC-HRT using published methods routine to the Organic Geochemistry Analysis Laboratory—GC × GC Facility at the Woods Hole Oceanographic Institution.^13,33–36^ For GC × GC-FID analysis, 1 μL aliquots of each sample were injected into a 310 °C splitless injector with a purge time of 0.5 min. The first-dimension column and the dual-stage cryogenic modulator are located in the main oven, while the second-dimension column is housed in a separate oven, enabling independent temperature control of all three components. The temperature program of the main oven was isothermal at 65 °C (12.50 min) and then ramped from 65 to 340 °C at 1.25 °C min^–1^. The second-dimension oven was programmed to remain isothermal at 70 °C (12.50 min) and then ramped from 70 to 345 °C at 1.25 °C min^–1^. The hot jet pulse width was 1.00 s, and the modulation period was 6.50 s with a 2.25 s cooling period between stages. The first-dimension column was a nonpolar Restek Rxi-1ms (59 m length, 0.25 mm I.D., 0.25 μm film thickness), and the second-dimension separations were performed on a 50% phenyl polysilphenylene-siloxane column (SGE BPX50, 1.25 m length, 0.10 mm I.D., 0.1 μm film thickness).

For GC × GC-HRT analysis, 1 μL aliquots of select samples were injected into a 310 °C splitless injector with a purge time of 0.5 min. The cold jet gas was dry N_2_ chilled with liquid N_2_. The hot jet temperature offset was 10 °C above the temperature of the main GC oven, and the inlet temperature was isothermal at 310 °C. The temperature program of the main oven was held isothermal at 60 °C (5 min) and was then ramped from 60 to 335 °C at 1.5 °C min^–1^. The hot jet pulse width was 2 s with a modulation period of 16 s. The second-dimension oven was held isothermal at 65 °C (5 min) and was then ramped from 65 to 340 °C at 1.5 °C min^–1^. The carrier gas was helium at a flow rate of 1 mL min^–1^. The first-dimension column was a Restek Rxi-5ms (30 m length, 0.25 mm I.D., 0.25 μm df), and second-dimension separations were performed on a Restek Rxi-17Sil MS (2 m length, 0.25 mm I.D., 0.25 μm film thickness). HRT data were sampled at an acquisition rate of 100 spectra per second in the mass range of 40–600 amu. The ionization method was electron ionization (EI) with an electron energy of −70 eV and an extraction frequency of 1.75 kHz. Chromatographic peaks were identified using pure standards or tentatively identified based on retention times in both dimensions, mass spectral matches (with a similarity above 80%; NIST/EPA/NIH 05 Mass Spectral Library), or mass spectral interpretation.^36^

### Fourier Transform Ion Cyclotron Resonance Mass Spectrometry (FT-ICR MS)

The FOPB-08 sample was analyzed by FT-ICR MS at the National High Magnetic Field Laboratory (NHMFL; Tallahassee, Florida). The sample was extracted into toluene/tetrahydrofuran/methanol (2:2:1) and analyzed by positive ion (+) atmospheric pressure photoionization (APPI) FT-ICR MS using a 9.4 T FT-ICR MS at the NHMFL.^37^

### Oil Weathering Simulations

The contributions of different oil weathering processes were constrained using a mass transfer model for the aqueous dissolution and evaporation of oil constituents.^38^ The model leverages aqueous solubilities and vapor pressures estimated for each pixel of a GC × GC-FID chromatogram.^39^ The GC × GC-FID chromatograms were baseline corrected using the algorithm of Eilers^40–42^ with specified parameters^43^ (lamda = 10^8^, *p* = 0.001, and *d* = 2) and normalized to the volume of the C_30_ hopane peak.^44^ Assuming equal contributions for dissolution and evaporation, the simulated water temperature was 30 °C and wind speed was 5 m s^–1^, and the simulation was run for 240 d. Simulations were initiated with the composition of the Brazil-12 sample^13^ (i.e., its GC × GC-FID chromatogram) run for six different initial oil layer thicknesses (0.33, 1, 3.3, 10, 33, and 100 μm). Oil was assumed to form a sea surface slick, dissolving on one side and evaporating on the other, with a constant surface area and decreasing thickness. The oil on the bottles was assumed to be a mixture of the six differentially weathered oils, which was fit to minimize the difference between the simulated chromatogram and the chromatogram of the samples (FOPB-01, -02, -05, -08, and -09). The fitting was performed over a defined rectangular region of the GC × GC chromatogram spanning from pixels 800 to 1400 in the first dimension and pixels 100 to 300 in the second dimension.

## Results

We assembled multiple lines of evidence to confirm our hypothesis of the transequatorial transport of spilled oil by floating debris. We investigated the following data sets in tandem: (1) arrival patterns of oil during the 2019 Brazil oil spill, (2) simulated back trajectories for the release of particles off the coast of Palm Beach (3) historical drifter experiments in the Western Tropical Atlantic and Caribbean Sea, (4) an analytical dispersion model based on the Okubo diagram, (5) long-term field observations of collected debris from Southeast Florida, and (6) molecular forensic comparisons of oily residues scraped from debris that arrived at Palm Beach in August 2020 to field samples of oil collected from Brazilian beaches in 2019. We show that ocean currents could transport the oiled debris. The timing of the unprecedented, extended arrival of oiled debris in Florida was consistent with the transport from the equatorial Atlantic Ocean following the 2019 Brazil oil spill, and the chemical composition of the oily residues from Palm Beach matched those from Brazil.

### Arrival of Oil along the Brazilian Coastline in 2019

The circumstances that led to oil contaminating ∼3000 km of the Brazilian coastline from August to December 2019 remain unsolved.^12^ However, details of the location and chronology of the oiling help explain the transport of the oil from Brazil to Florida. Oily residues collected in 2019 from the impacted Brazilian coastline, between latitudes 20° S and 3° S, shared the same source.^13–15,45^ Because oiling was found north and southward of the western boundary currents system bifurcation (14° S average position), the release point of the oil would have been within or near the southern branch of the South Equatorial Current.^46^ The latter allowed the spilled oil to be transported north and west along the continental slope by the North Brazil Current and south by the Brazil Current. It is reasonable that some fraction of the oil remained in the North Brazil Current and was then carried by the Guyana Current and the Caribbean Current into the Loop Current of the Gulf of Mexico, through the Florida Straits, and finally stranded along the southeastern Florida coastline (Figure 2). Presumably, the oil encountered marine debris at some unknown point during this transit, with a greater probability of interaction closer to Brazil than farther away due to weathering and/or engineered spill response measures.

### Numerical Modeling of Possible Trajectories

Oceanographic simulations supported the hypothesis that the oiled debris could have originated off the coast of Northeast Brazil. The OpenDrift model^22^ with currents data from the GLORYS12 V1 Global Ocean Physics Reanalysis^23^ was used to calculate one-year backward trajectories of 10,000 particles arriving at Palm Beach, Florida between May and August 2020. The modeling showed that particles can originate from a large ocean region, including several locations within the Gulf of Mexico and extensive areas off the coasts of Central America, as well as North and Northeast Brazil (Figure 3). The latter regions coincide with the probabilistic trajectories of oil released during the 2019 Brazil oil spill, supporting their likelihood as the source of the debris.^47^ Considering the area subjected to the Brazilian oil spill event (Figure 2), debris floating in the region under the influence of the North Brazil Current and the Central Branch of the South Equatorial Current in 2019 could arrive in the Palm Beach region in one year or less. The main path of the particles toward Southeast Florida was the North Brazil Current, followed by the Guyana Current, Caribbean Current, and Loop Current (Figures 2 and 3).

**Figure 3:**
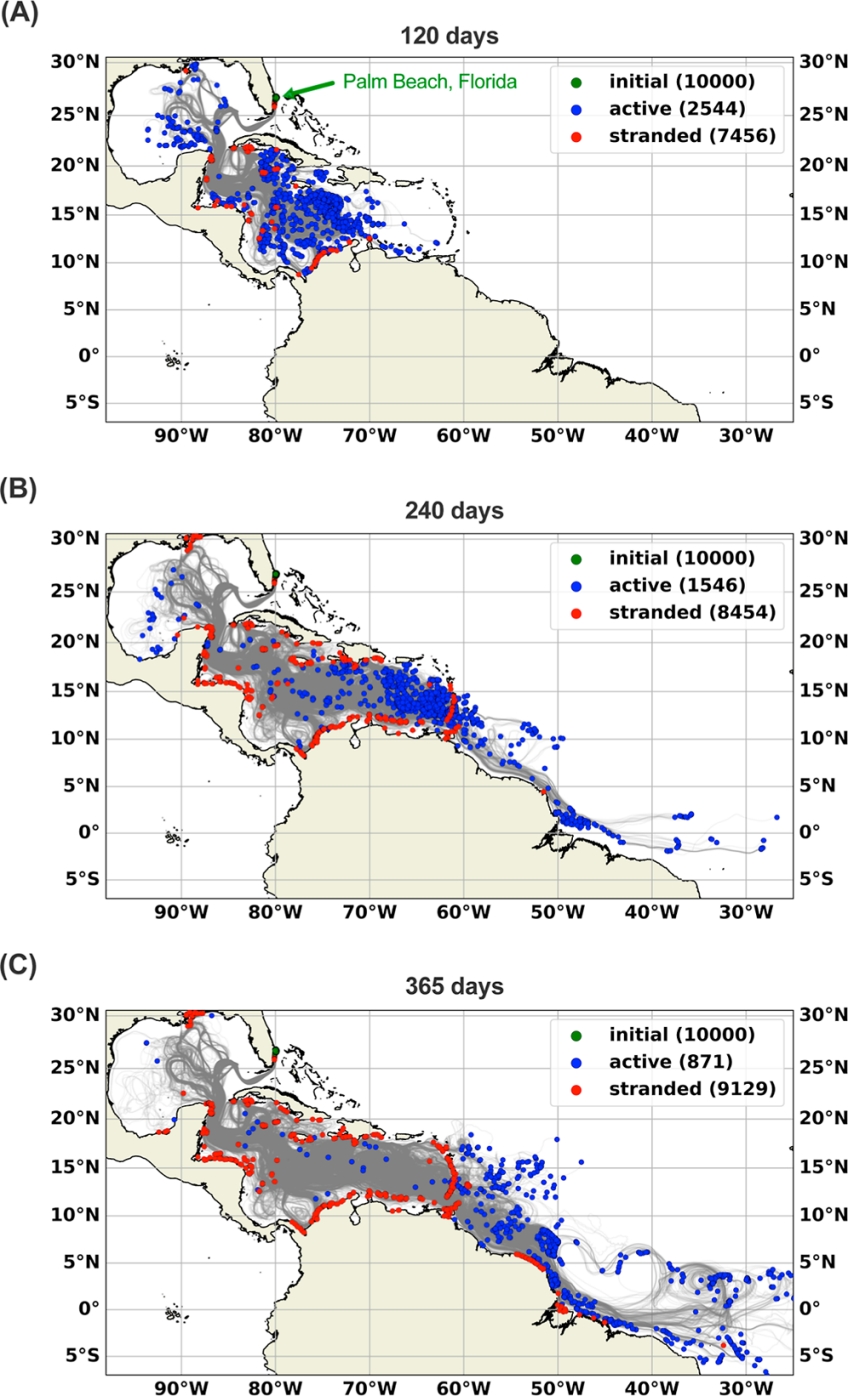
Numerical modeling of possible spill trajectories. Spatial distribution of the backward trajectories of 10,000 particles released in Palm Beach, Florida (green point) within 120 d (A), 240 d (B), and one year (C) of release. Particles were released from August to May and tracked backward for one year. Red dots (stranded) denote particles that originated from stranding on land. Blue dots (active) denote the final positions of particles remaining at sea. The gray lines highlight the possible paths the debris may have taken. Each gray line corresponds to the trajectory of a single particle.

### Historical Drifter Experiments

Due to the similar size and shape,^48^ drift-bottle experiments focusing on the surface currents of the Western Tropical Atlantic and Caribbean Sea in the 1960s and 1970s provide excellent context and reference to the transequatorial transport of the oil-covered bottles to Southeast Florida.^48–50^ Drift bottles released along an array of stations north of Northeast Brazil were recovered in Southeast Florida after 133 to 402 d, having traveled 4600 to 5700 km at an estimated velocity of 14 to 37 km day^–1^.^49^ One drift bottle released in the Grenada Passage was found 76 d later in Boynton Bay, Florida (several km south of Palm Beach), equivalent to a 46.5 km day^–1^ velocity.^50^ Brucks^48^ reported monthly surface velocities in the Caribbean Sea from drift bottle experiments to range from 9.8 to 37 km day^–1^, with a mean of 23 km day^–1^ in 1967 and 1968. High-resolution monitoring of satellite-tracked drifters has recorded similar trajectories and current velocities from the North Brazil Current through the Florida Current.^51^ With an estimated 240-d transit to Florida, traveling ∼8500 km, the surface velocity of the oil-covered bottles was ∼35 km day^–1^.

### Dispersion of Surface Floating Particles

Modeling the dispersion of floating objects reinforced the particle backward trajectory evidence supporting the transequatorial transport of oiled debris from Brazil to Florida. For these analyses, we considered the dispersion of a cloud of particles during their transport from offshore Brazil to Southeast Florida. We assumed the floating debris interacted with a localized and quasi-instantaneous spill and then spread as they moved toward Florida with ocean currents. Drifter experiments have reported that travel times from the equatorial Atlantic to Southeast Florida can range from 150 to 400 d. Using the Okubo diagram,^31^ which relates apparent diffusion to cloud size, we estimated that debris would spread out to a large enough patch size (i.e., estimates of the blue curve in Figure 1C) that the debris may have taken between 20 d (under the faster transport speeds) and 300 d (under the slower speeds) to pass Palm Beach. This time range is consistent with reports that debris washed ashore from late May to September 2020, a period of ∼130 days (Figure 1C). Applying the Okubo diagram to determine a travel time corresponding to 130 days of contamination, we found 240 days, or about eight months, to be the travel time of the patch of debris between the oiling of Brazilian beaches and the peak of beach contamination in Florida. This period aligns with the time between the Brazil oil spill and the arrival of oil-covered debris in Florida (Figure 1C).

### Long-Term Observations from Beach Clean-Ups on Southeast Florida Beaches

While oiled debris is occasionally collected, the FOPB had never observed an oiling event of similar magnitude. Since 2015, members of the FOPB have surveyed and hand-collected debris along 12 km of beach every Monday through Friday of the year. The mass of debris collected ranges from 13,500 to 23,200 kg yr^–1^. The high-frequency surveys by the FOPB, along with their awareness of the debris type, have established a seasonal baseline capable of identifying and investigating singular arrivals and short-term debris events. For example, on July 24, 2019, the FOPB found a diving safety device with the owner’s contact information. The device was lost in Cozumel, Mexico, on July 4, 2019, and returned to the owner. Another example was in late October and through December 2016, when Palm Beach was inundated with hundreds of used drinking water pouches (120 and 240 mL in size). The FOPB connected the pouches to relief efforts in Haiti following the October 4, 2016, arrival of Category 4 Hurricane Matthew. These two examples of “drifters of opportunity”^52^ align with historical drift-bottle data and the back trajectory modeling results (Figure 3).

Unlike the diving safety device and water pouches, the oiled debris that arrived in Palm Beach was diverse and typical of the nonoiled debris collected by the FOPB, ranging in material type, size, shape, color, product usage, and physical wear (Figure S1). Single-use plastic beverage bottles without wrapper labels were the most abundant, followed by smaller and larger plastic bottles. Direct print and embossed tops, and distinct shapes helped identify some of the plastic bottles as those used for personal care products, dietary supplements, and fuel or antifreeze. Glass bottles typical of wine and liquor were often found. All containers were capped. Unidentifiable pieces of expanded foam were also frequently encountered, some of which had black residues. Despite the uptick in the number of oiled debris items arriving in the summer of 2020, most of the debris that washed ashore was not oiled.

### Molecular Forensics of 2019 Brazil and 2020 Florida Samples

Comprehensive molecular-level analytical methods developed for oil spill forensics provided definitive evidence for the transequatorial transport of oiled debris. With the high cost of fines, cleanup, and restoration to impacted areas following an oil spill, advanced (and legally defensible) approaches have been developed to determine if oily samples are from a suspected source of oil (e.g., tanker, pipeline, or platform).^53^ Compared to a suspected source, an oily sample is a “match” if the loss of the more labile hydrocarbons is consistent with the level of environmental exposure and the more stable hydrocarbons are quantitatively unchanged, with the ratios of stable compounds being statistically similar.^54,55^

Analyses of the black residues scraped from six FOPB samples (Table S1; Figure S2) by GC-FID and GC–MS, GC × GC-FID and GC × GC-HRT, and FT-ICR MS revealed a complex mixture of petroleum hydrocarbons (Figure 4; Tables S3–S5; Figures S3–S6).^56–58^ These samples were compared to two representative oil samples from the 2019 Brazil oil spill (Brazil-12 and Brazil-12/13) that have been previously reported.^13^ All samples compared were analyzed using the same methods and laboratories (Table S2).

**Figure 4:**
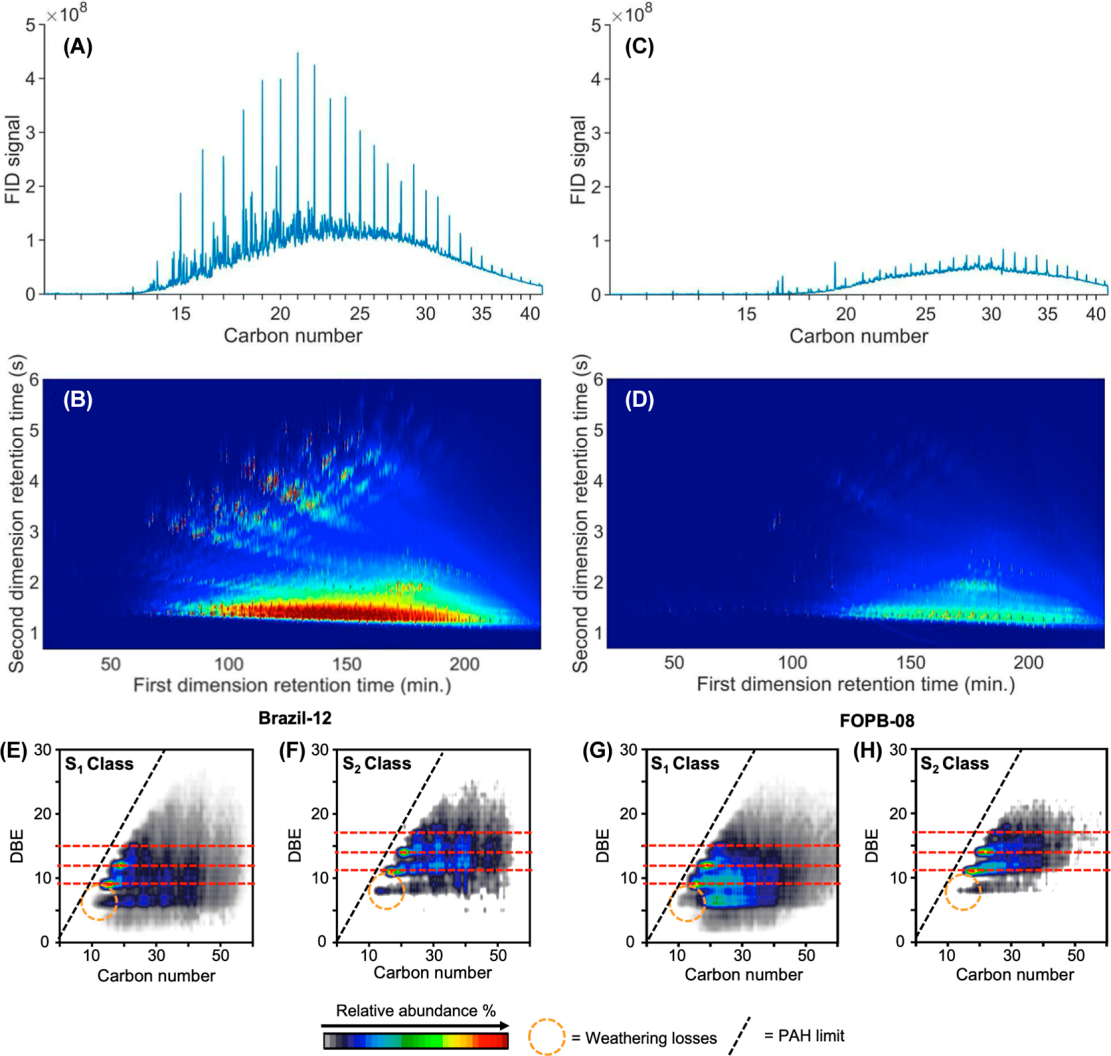
Chemical analysis of black residues. Reconstructed one-dimensional GC-FID chromatograms from GC × GC-FID analyses and GC × GC-FID chromatograms of the Brazil-12 sample (A,B) and the FOPB-08 sample (C,D), respectively. Isoabundance contour plots of double bond equivalents (DBE, defined as the number of rings plus double bonds to carbon) versus carbon number for S_1_ and S_2_ species, that is, compounds containing C, H, and one or two S atoms of the Brazil-12 sample (E,F) and the FOPB-08 sample (G,H), respectively. The data presented in panels E–H were obtained via (+) APPI FT-ICR MS.

### Molecular Features Indicated a Refined Product

The gas chromatograms of the FOPB samples revealed a complex mixture of alkanes eluting from C_20_ to C_40_ with varying amounts of *n*-alkanes and other resolved peaks (Figure 4; Figure S3). Samples FOPB-01, -05, −08, and −09 were more closely related (Figures S3–S6). Sample FOPB-02 contained an additional hump featuring C_15_ to C_22_ *n*-alkanes. Sample FOPB-03 was unlike the others (Figure S7); it was fresher in composition than the 2019 Brazil samples, thereby making the Brazil oil spill an unlikely source. Chlorinated and brominated compounds were not detected in the oils. Like the 2019 Brazil oil samples, the presence of 2-methylanthracene (Table S3), a product of petrochemical refining, removed any possibility that the FOPB residues were from accidental releases of crude oil or naturally seeped oil.^13^

The FOPB-08 sample was analyzed by (+) APPI FT-ICR MS, yielding ultrahigh-resolution mass spectra that enabled the assignment of unique molecular formulas to thousands of detected peaks based on mass accuracy. Compared to the Brazil-12 sample, both residues exhibited a highly similar aromatic “signature”. Extensive studies in petroleum geochemistry have demonstrated that the distribution of aromatic core structures, particularly compounds with one or two sulfur atoms (S_1_ and S_2_ classes) (Figure 4E–H; Section S1) is highly sensitive to thermochemical refining.^13,59,60^ The observed similarity in these distributions between the two samples provides compelling evidence that both residues probably share a common thermal processing history. This finding supports the GC–MS and GC × GC-FID data, reinforcing that both residues likely originated from the same refined source despite differing environmental alterations.

### Oil Weathered as Expected

Based on existing knowledge of oil fate when spilled at sea, it was expected that the FOPB samples would preferentially lose labile and enrich recalcitrant chemical constituents when compared with oil from the 2019 Brazil oil spill due to their relative stability to the combined effects of weathering processes (evaporation, dissolution, photochemical degradation, and biodegradation). Compared to the Brazil-12 sample, the FOPB-01, -02, -05, -08, and -09 samples lost 54–72% of their GC × GC-amenable mass. The losses comprised many of the resolved peaks, including hydrocarbons that eluted earlier than C_18_ and polycyclic aromatic hydrocarbons (PAHs) (Figures S4–S6). To better constrain the contributions of the different weathering processes to the observed changes (Figure 4), a mass transfer model for evaporation and aqueous dissolution of oil constituents was applied.^38^ Modeling these processes revealed that 40–50% of the GC × GC-amenable mass of the Brazil-12 sample could have been lost through evaporation and aqueous dissolution alone (Figures S8–S12). As a result, an additional 14–27% of the GC × GC-amenable mass of the Brazil-12 sample was assumed to have been lost to biodegradation and photodegradation (Figures S8–S12). Overall, diagnostic ratios of the alkanes and PAHs reflected the outcomes of the modeling (Section S2; Figure S13). The observed pattern of losses is consistent with the known lability of compounds to weathering processes, with preferential losses of *n*-alkanes and PAHs, and enrichment of petroleum biomarkers that were used to fingerprint the samples.^38,42,43,61,62^

### Biomarker Fingerprints Matched

Petroleum biomarkers, chemical remnants of organisms found in crude oil, provide sensitivity and selectivity for characterizing oils and differentiating oil-contaminated samples from potential sources.^63^ The distribution and relative abundance of the recalcitrant biomarkers (hopanes, steranes, and diasteranes) in the FOPB-01, -05, −08, and −09 and the Brazil-12 sample exhibited similar GC × GC chromatograms, including a unique distribution of C_20_ to C_25_ tricyclic terpanes, C_24_ tetracyclic terpane, benzohopanes, diginane, 25-norhopanes, and 8,14-secohopanes (Figure 5; Figures S14–S17).^13^ Qualitatively, the biomarker region of FOPB-03 did not match that of the other samples (Figures S6 and S7), requiring further investigation to determine its source. From detailed quantitative analysis, 12 “environmentally stable” diagnostic ratios of biomarkers in the FOPB-01, -05, -08, -09, and Brazil-12/13 samples were statistically equivalent (Figure 5E).^55^ Conversely, the FOPB-01, -05, -08, and -09 samples did not match oil from numerous current and historical sources from the Gulf of Mexico region, including the 2010 *Deepwater Horizon* disaster, two other oil spills (Mud Lake and West Delta 117 spills), three production crude oils (Horn Mountain, Dorado Field, and King West Field) (Figure 5F), and several naturally seeped oils (Green Canyon, Heron’s Mound, Mobile Dome, Farnella Dome, Horn Dome, Dauphin Dome, and Biloxi Dome) (Figure 5G).^55^

**Figure 5:**
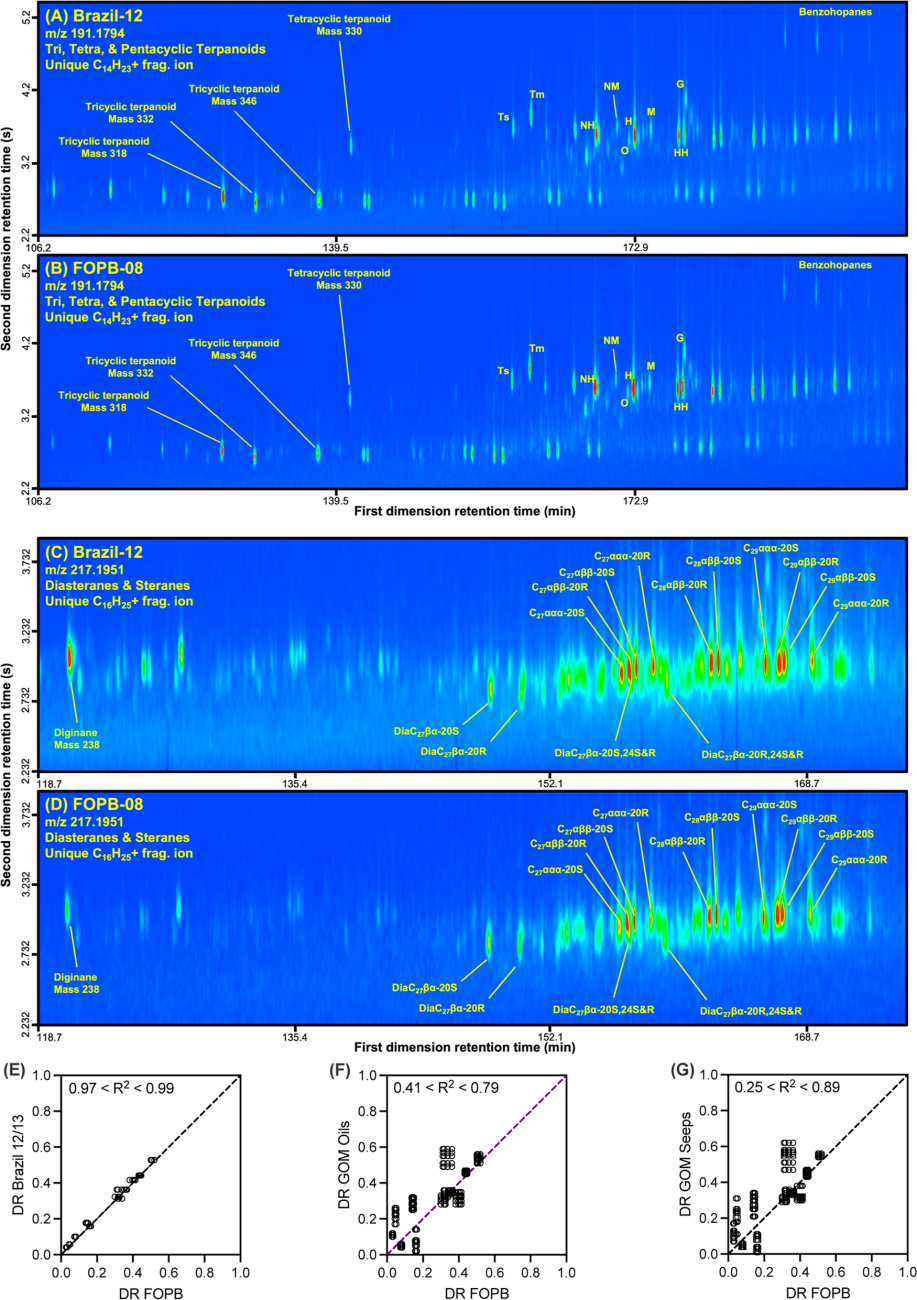
Petroleum biomarker fingerprinting. The extracted ion signal from the GC × GC-HRT for the hopanoid biomarkers (*m*/*z* 191.1794) for (A) the Brazil-12 and (B) the FOPB-08 samples and disasteranes/steranes (*m*/*z* 217.1951) for (C) the Brazil-12 and (D) the FOPB-08 samples. Comparison of 12 diagnostic ratios (DRs) of petroleum biomarkers categorized by Stout^55^ as “excellent” for relative stability during weathering between the FOPB samples (FOPB-01, -05, -08, -09) and the (E) Brazil-12/13 oil and, reported values for (F) Gulf of Mexico (GOM) oils and spills, and (G) natural seeps. The extent of the linear correlation between DRs for the different samples provides evidence that they are from the same source.

In summary, several of the FOPB samples (FOPB-01, -02, -05, -08, and -09) matched the Brazil oil samples, but not all (FOPB-03). We concluded this because the petroleum hydrocarbons expected to weather in transit from Brazil to Florida were not present, and those recalcitrant to weathering on the time scale of the transit were present in ratios like those in the oil residues collected along the Brazilian coast in 2019.

## Discussion

We tested the unprecedented hypothesis that oil adhering to the debris littering the southeastern Florida coastlines originated from offshore Brazil, having traveled ∼8500 km via surface currents and arrived in ∼240 days. Physical oceanography provided plausibility for this hypothesis, which was verified by molecular forensics of the oil.

### No Other Reports of Oiled Debris in the Region

While dispersion modeling indicated that oiled debris would have reached other Caribbean countries (Figure 3), no records indicate this occurred. A review of drift bottle experiments that were conducted from 2001 to 2018 in the North Atlantic Ocean found that the predicted and actual locations with the highest bottle return differed.^64^ The discrepancy was attributed to biases in population density, coastal access, and lifestyle of neighboring communities. Presumably, decreased tourism, curfews, and other factors resulting from COVID-19 lowered the likelihood of observing the 2019 oil spill event.^65^ As for the 2020 oily debris event, the coarrival of *Sargassum* along the coastlines may have masked it.^66^

We suspect that the FOPB detected the oily debris because of its long-term, regular cleanup of marine debris over a fixed section of beachfront, effectively acting as a coastal monitoring station with a sensitivity for such events. The FOPB focuses on cleaning macroscopic debris, likely letting smaller pieces go unnoticed. Owing to the vast size range of marine plastic debris (nm to m),^67^ presumably, many smaller pieces of plastic were oiled and evaded detection at Palm Beach and at any other location. Some oiled debris was likely carried to the North Atlantic gyre and elsewhere or sedimented to the seafloor.^68^

### Detecting Long-Range Transport of Oil is Unprecedented

Oil rarely travels more than 300 km following an oil spill^18^ because of the combined effects of weathering, entrainment, emulsion, and dispersion.^69^ A notable exception was the *Ixtoc I* spill, which released 492 million liters of crude oil over 290 days into the Gulf of Mexico in 1979.^70^ Two months after the start of the spill, approximately 11 million liters of oil had traveled nearly 1000 km from the source in the Bay of Campeche to the Texas coastline (Figure 2).^20,71^ This outlier can be attributed to the nature of the spill, which was a sustained release of a large quantity of oil from a point source at a relatively shallow depth (∼60 m). The long-range transport of oil from Brazil to Florida differed in the magnitude of the distance traveled (∼8500 km), presumably because it traveled by adhering to floating marine debris.

The co-occurrence of oil and floating debris from disasters and accidents has been documented, though transport generally remains local (<250 km).^72,73^ However, with numerous studies documenting the long-range transport of acute releases of “drifters of opportunity,”^52^ such as rubber duckies,^74^ Nike sneakers,^75^ and inkjet cartridges,^76^ it is reasonable that oily residues sorbed to floating debris can travel very long distances,^77^ The extent to which buoyant marine debris protects adherent oil from the conventional processes (i.e., weathering, entrainment, emulsion, and dispersion) that limit the long-range transport of free-floating oil requires further study.

### Oiled Plastic is a Globally Occurring Potentially Hazardous Cocontaminant

Oiled plastics found on beaches have been reported since the 1970s but have remained an understudied type of marine pollution.^78^ Historically, these cocontaminants have been referred to as plastitar,^79^ plasto-tarball,^80^ plasto-tar-crust,^80^ or petroplastic.^72^ The hydrophobic and rough surfaces of marine plastics can facilitate the adsorption of hydrophobic oil.^81,82^ Leveraging this feature, the International Pellet Watch (IPW) developed an approach for monitoring oil pollution by measuring petroleum biomarkers (i.e., hopanes) associated with beached and floating resin pellets.^83^ Beyond the measurements made by the IPW, little is known about the source(s) and type of petroleum hydrocarbons on this form of marine debris.^72^

Many properties of the plastic and the oil can be expected to differ because of their association. The interplay of oil and floating debris may affect the fate and transport of each contaminant,^81,84^ namely from changes in the density of the composite. The weathering of plastics and associated oils by mechanical, microbial, and photochemical processes^85^ are expected to be altered by the characteristics of the oil (sweet to sour; light to heavy; crude to refined).^86^ The extent to which the layer of oil protects the underlying plastic from weathering processes warrants investigation. Petroleum residues can also be stronger sorbents than polyethylene for other hydrophobic organic contaminants and, hence, may house a greater proportion of potentially toxic chemicals than unoiled plastic.^87,88^ However, we did not detect common halogenated legacy pollutants (e.g., polychlorinated biphenyls, PCBs, and dichlorodiphenyltrichloroethane, DDT) or other compounds in the oil residues often concentrated by marine plastics.

While surveys of oil and plastic pollution have been ongoing for decades,^89,90^ comparable surveys for oiled or tarred plastic are nonexistent. Beginning in the mid-1950s,^3^ numerous studies have monitored the presence of floating oil (or tar) in the world’s oceans.^90,91^ Today, hotspots for oil slicks are located around natural seep zones, areas of offshore oil and gas development, and shipping routes.^92^ Along with the records of floating tar and oil slicks, a similar record exists for floating plastic.^89,91^ Currently, numerous global hotspots for oil slicks also overlap with areas of elevated marine plastic debris concentrations and their primary input sources,^93,94^ increasing the global occurrence of oiled plastic in the ocean.

We showed that marine debris can facilitate the long-range transport of oil and suggest that oil, in turn, may impact the transport of marine debris. While the origin of most marine debris is unknown, the identity of the associated oil provided a rough starting location for the floating debris and a chronometer to back out its travels. Yet, much remains to be understood regarding how oil sorbed to debris affects their collective transport, fate, and toxicity. To begin addressing these uncertainties, beach and ocean surveys will need to specifically monitor for oiled plastic debris and provide real-world data to quantify the extent of this likely prevalent form of pollution.

### One World Ocean

This study reminds and encourages individuals and organizations (e.g., FOPB) that scientists do not need specialized advanced degrees and professional affiliations but rather a willingness to remain curious, investigate, and communicate their observations and findings.^95^ In 1942, Athenius Spilhaus argued that the “world ocean is a continuous body of water” and proposed a new approach to mapping oceanographic features that highlights the oceans rather than continents.^96^ More recently, nonprofit organizations and government entities have embraced Spilhaus’s concept to promote awareness of the ocean’s connectedness and that humans only have one ocean.^97^

With a shared interest in understanding the sources and movement of marine pollution, the knowledge-sharing and collaborative nature of this international investigation by academic and nonacademic stakeholders led to a reasonable and rational explanation for a singular event. The results of this unique study underscore the concept of the one world ocean, reminding society that local inputs of pollutants into the ocean can have transboundary impacts.

## Supplementary Material



## Data Availability

All data are available in the main text or the Supporting Information.

## Notes

Discussion of sample shared thermal history (Section S1); discussion of PAH diagnostic ratios (Section S2); photos of oiled debris in the field (Figure S1); photos of oiled debris samples (Figure S2); GC-FID chromatograms (Figure S3); saturated hydrocarbon distributions (Figure S4); PAH distributions (Figure S5); GC × GC-FID chromatograms (Figure S6); GC × GC-FID chromatograms of FOPB-03 (Figure S7); modeling of oil weathering (Figures S8–S12); PAH diagnostic ratios (Figure S13); GC × GC-HRT extracted ion chromatograms (Figures S14–S17); sample information (Table S1); analyses performed for each sample (Table S2); saturated hydrocarbon contents (Table S3); PAH contents (Table S4); petroleum biomarker contents (Table S5); additional references (PDF)
