# Supplementary material for: Long-Range Transport of Oil by Marine Plastic Debris: Evidence from an 8500 km Journey
Source: Environ Sci Technol. 2026 Jan 7;60(3):2649–61. doi: 10.1021/acs.est.5c14571 (PMC12854741; doi:10.1021/acs.est.5c14571)
Supplement: Supplementary file 1 [file es5c14571_si_001.pdf]

Supporting Information

Long-range transport of oil by marine plastic debris: Evidence from an 8,500 km journey

Bryan D. James[1,2]*, Luis E.A. Bezerra[3], Diane Buhler[4], Rivelino M. Cavalcante[3], Martha Liliana Aguilera[5], Bing Chen[6], Jonas Gros[7], Ulrich M. Hanke[2], Karin L. Lemkau[8], Robert K. Nelson[2], Sydney F. Niles[5,9], André Henrique Barbosa de Oliveira[10], Thomas D. Pitchford[4], Jagoš R. Radović[11], Ryan P. Rodgers[5,9], Marcelo O. Soares[3], Scott A. Socolofsky[12], Roger E. Summons[13], Robert F. Swarthout[14], Carlos E. P. Teixeira[3], David L. Valentine[15,16], Helen K. White[17], Min Yang[6], Eliete Zanardi-Lamardo[18], Baiyu Zhang[6], Christopher M. Reddy[2]*

Corresponding authors: creddy@whoi.edu; b.james@northeastern.edu

Number of Pages: 26
Number of Figures: 17
Number of Tables: 5

Section S1. Extended discussion of the shared thermal history of the oil samples

For the S_1 class (**Figure 4E and 4G**), the dominant series at double bond equivalent (DBE) values of 9, 12, and 15 indicated a prevalence of dibenzothiophenes, naphthodibenzothiophenes, and dinaphthodibenzothiophenes, respectively, which are known as thermally stable sulfur-containing aromatic cores in petroleum. The clustering of these compounds at low carbon numbers, near the PAH limit (**Figure 4E-H; black dashed line**), suggests exposure to thermal cracking and reflects a shared thermal history. In the Brazil-12 sample (**Figure 4E-F**), sulfur-containing aromatics were particularly abundant near the PAH limit, whereas in the FOPB-08 sample (**Figure 4G-H**) there was a relative depletion of lower-carbon-number species, consistent with extended weathering.

Section S2. Discussion of diagnostic ratios of saturates and PAHs indicative of weathering process

Diagnostic ratios of alkanes and PAHs are used to quantitatively assess the extent of weathering of an oil. Ratios and their values have been developed to indicate the extents of dissolution, evaporation, biodegradation, and photodegradation. While a single diagnostic ratio can be imperfect, together they offer insights into understanding the fate of the oil in the environment. Ratios of alkylated napthalenes, alkylated phenanthrenes, and alkylated dibenzothiopenes can indicate the extent of dissolution.[5,6] Ratios using phenathrenes and dibenzothiophenes indicated similar extents of dissolution may have occurred for each of the FOPB samples (**Figure S13**).[7] Other ratios for dissolution and evaporation[5] using naphthalenes and *n*-alkanes were indeterminate (**Figure S13**) because these compounds either were already depleted in the Brazil oil or depleted in the FOPB samples. Ratios for biodegradation include ratios of specific linear to branched alkanes and alkylated dibenzothiophenes.[8,9] The ratios of *n*-heptadecane to pristane and *n*-octadecane to phytane indicated biodegradation for most of the FOPB samples but were indeterminate for others suggesting less biodegradation than the Brazil mystery oil (**Figure S13**). This discrepancy could be from contamination from natural and/or anthropogenic background sources of these compounds. Comparatively, ratio of the alkylated dibenzothiophenes indicated biodegradation for all the FOPB samples (**Figure S13**). Lastly, ratios for photodegradation include those between benz[*a*]anthracene to chrysene, phenanthrenes, and chrysenes.[5,10] As with the other ratios, some indicated photodegradation while other were indeterminate (**Figure S13**).

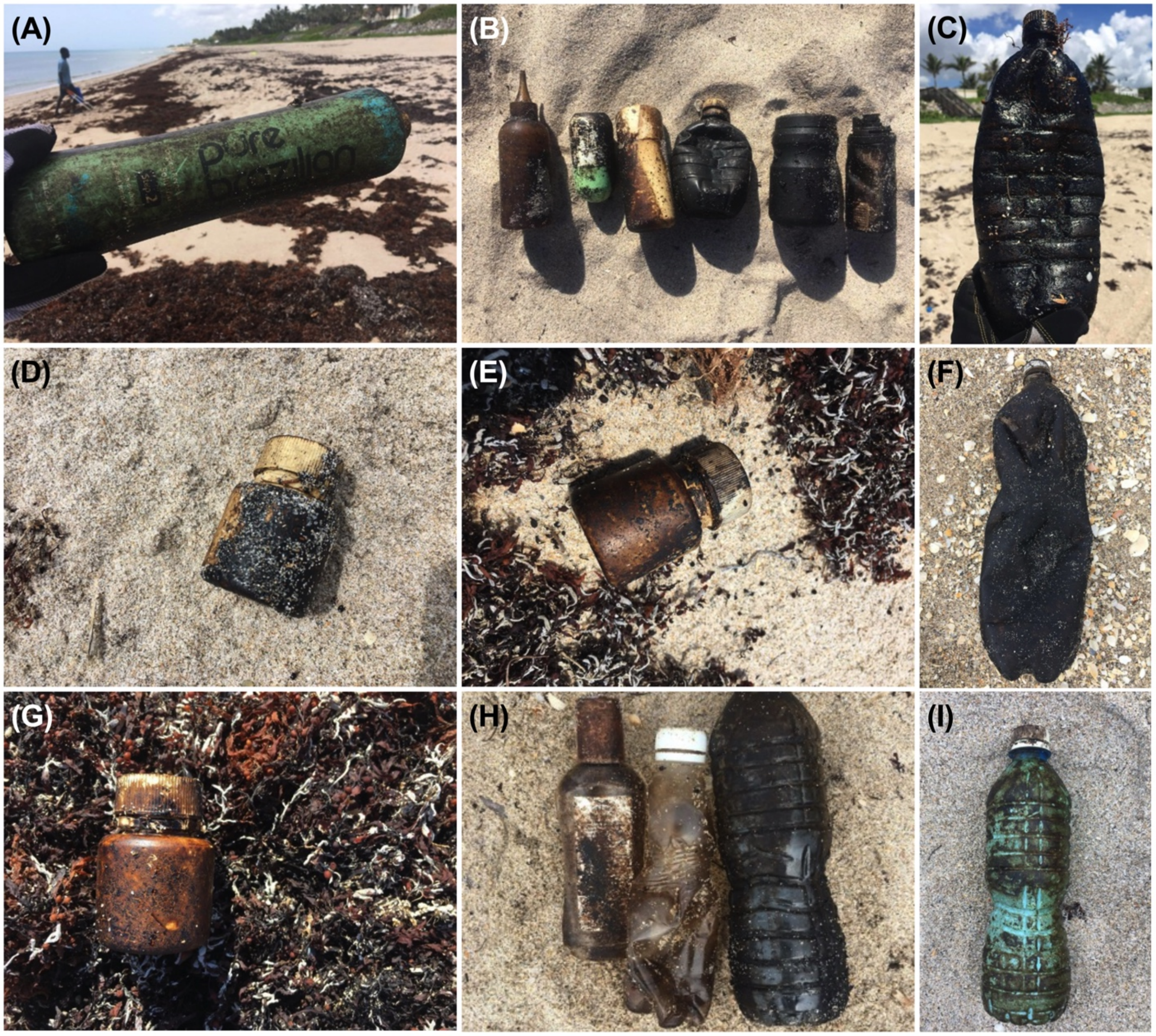

Figure S1. Photographs of oiled debris collected by the FOPB in the summer of 2020.

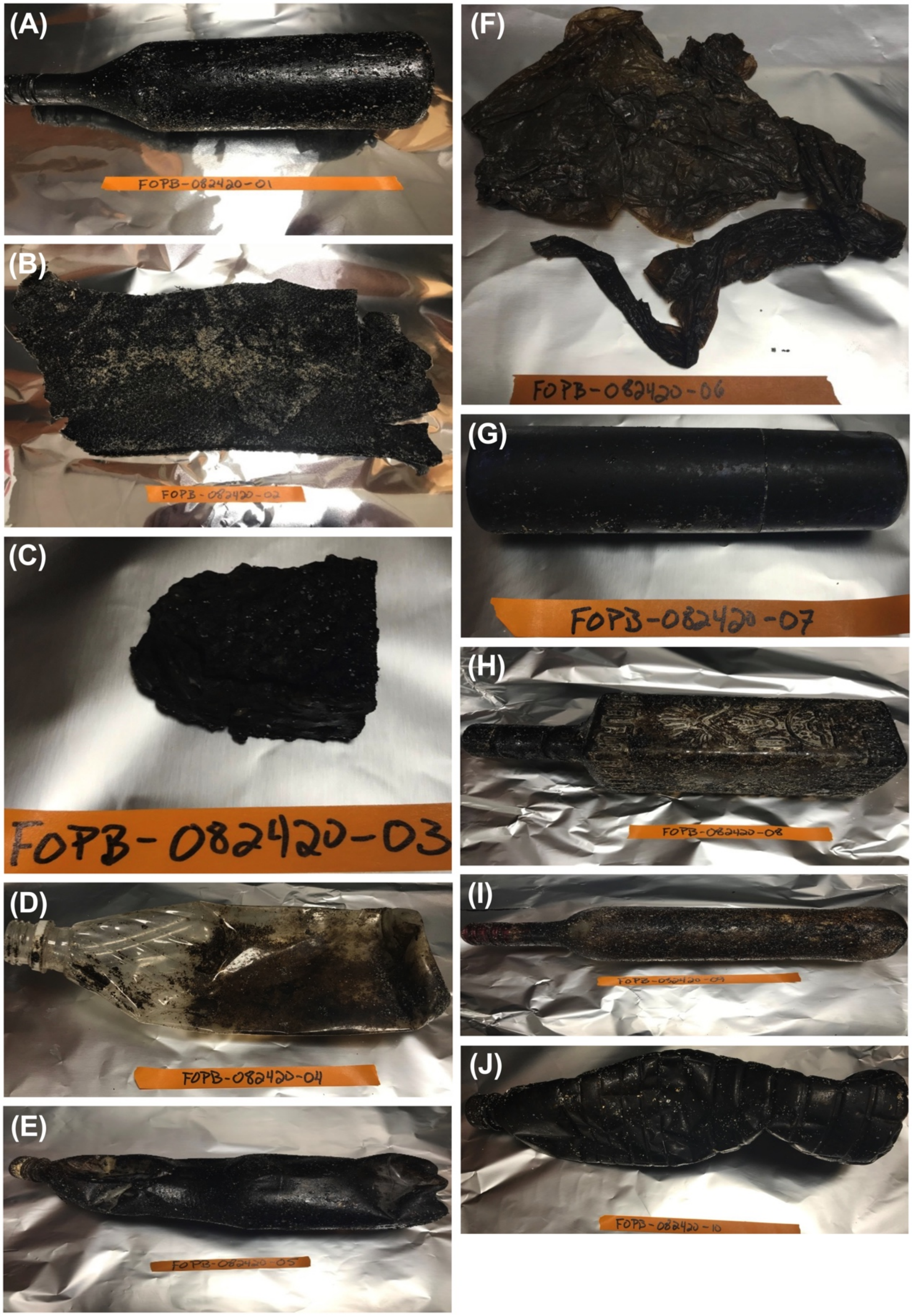

Figure S2. Photographs of samples (A) FOPB-01, (B) FOPB-02, (C) FOPB-03, (D) FOPB-04, (E) FOPB-05, (F) FOPB-06, (G) FOPB-07, (H) FOPB-08, (I) FOPB-09, and (J) FOPB-10.

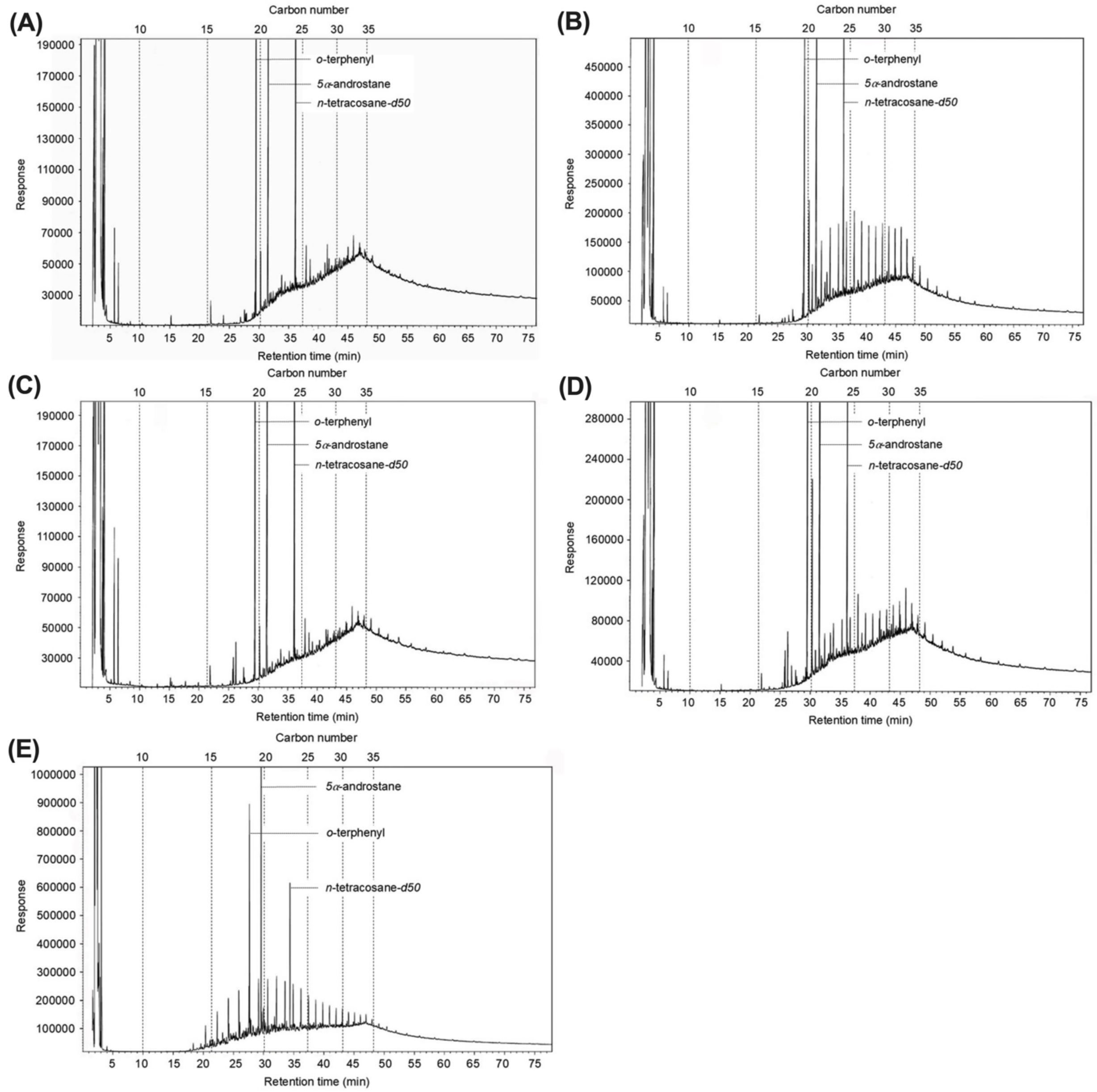

Figure S3. GC-FID chromatograms of (A) FOPB-01, (B) FOPB-05, (C) FOPB-08, (D) FOPB-09, and (E) Brazil-12/13. Labeled peaks belong to added standards 5α-androstane, *o*-terphenyl, and *n*-tetracosane-*d*50.

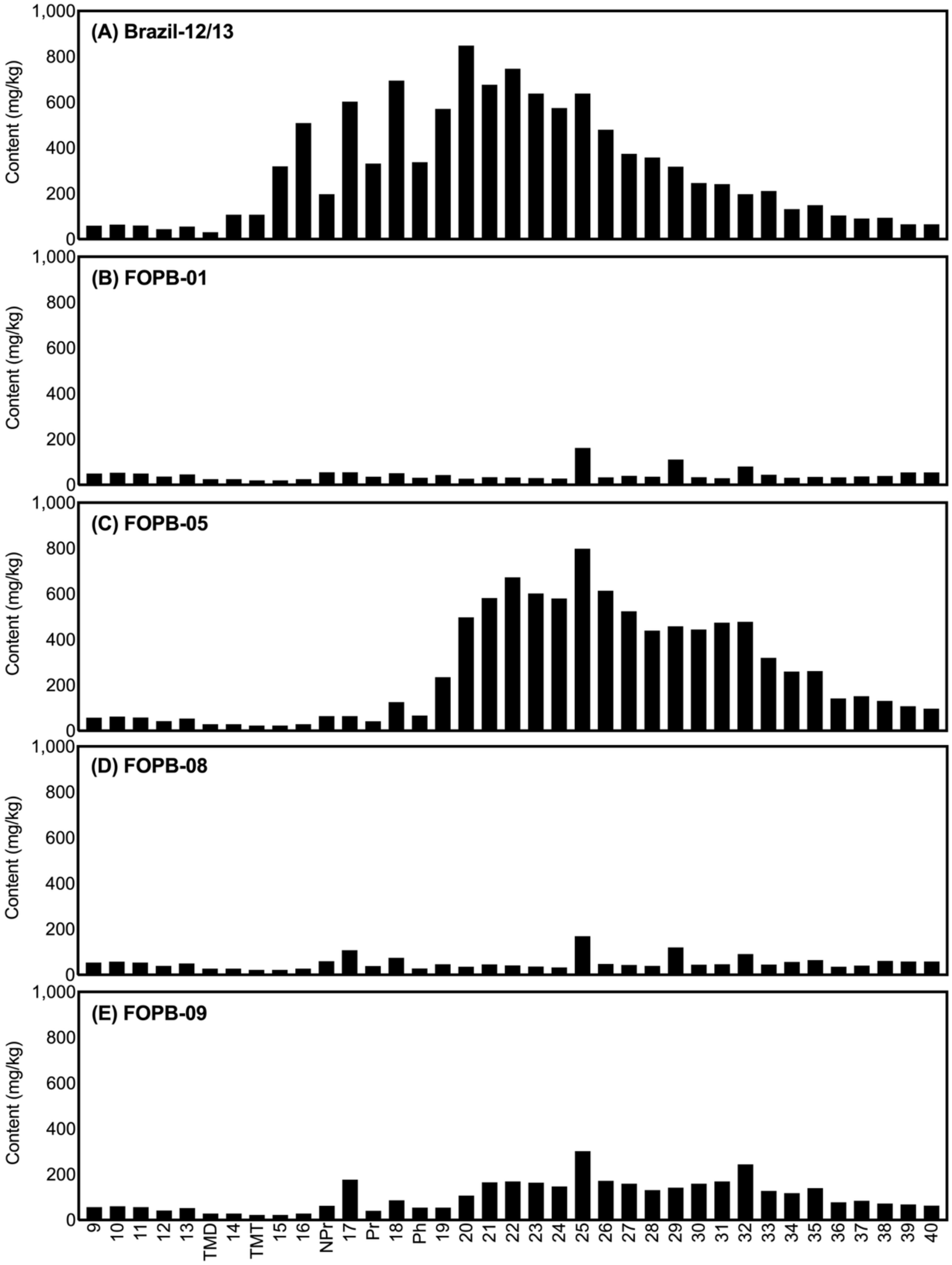

Figure S4. *n*-alkanes and select branched alkanes in (A) Brazil-12/13, (B) FOPB-01, (C) FOPB-05, (D) FOPB-08, and (E) FOPB-09. The horizontal axis labels correspond to *n*-alkanes of 9-40 carbon number and 2,6,10-trimethyldodecane (TMD), 2,6,10-trimethyltridecane (TMT), norpristane (NPr), pristane (Pr), and phytane (Ph) are branched alkanes.

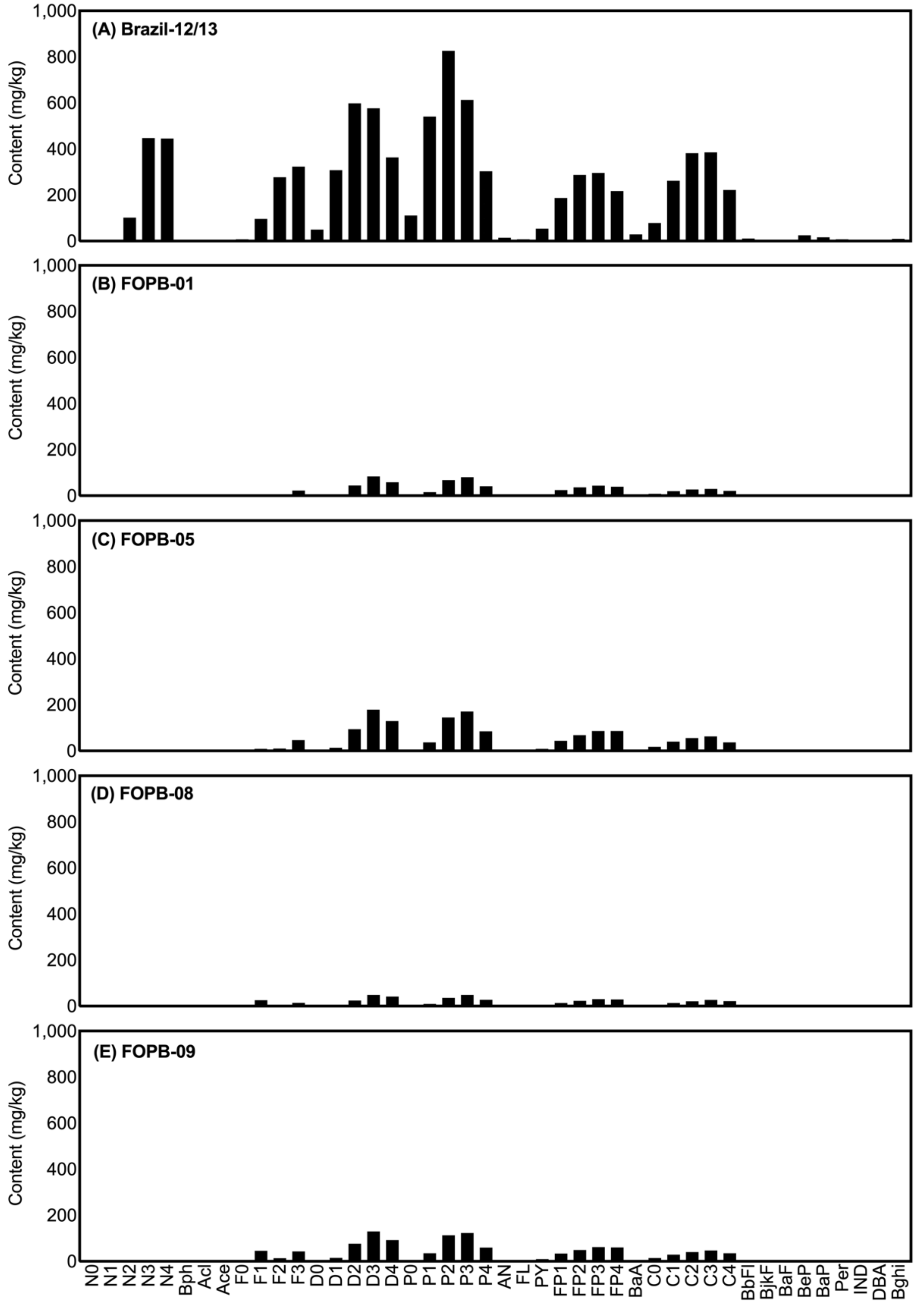

Figure S5. Parent and alkylated PAHs in (A) Brazil-12/13, (B) FOPB-01, (C) FOPB-05, (D) FOPB-08, and (E) FOPB-09. Acronyms are specified in Table S3.

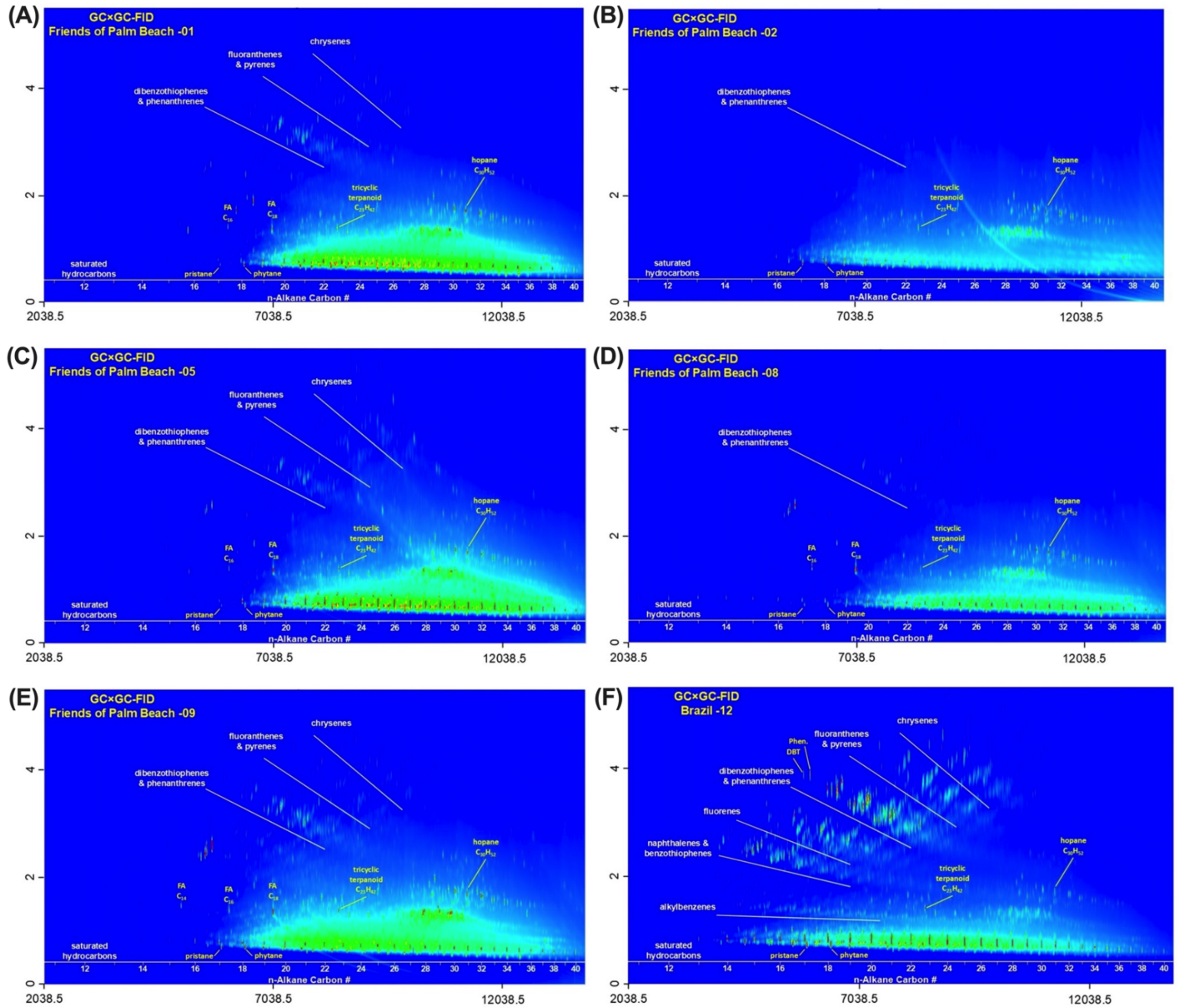

Figure S6. GC×GC-FID chromatograms of (A) FOPB-01, (B) FOPB-02, (C) FOPB-05, (D) FOPB-08, (E) FOPB-09, and (F) Brazil-12.

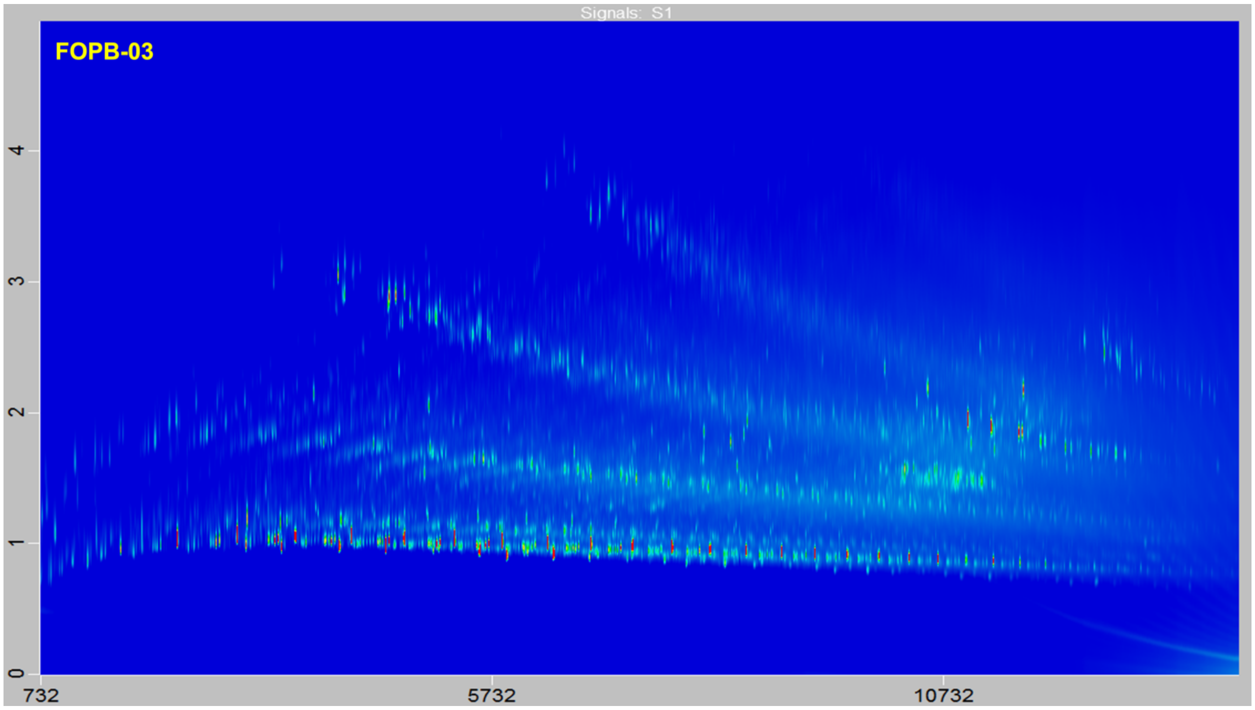

Figure S7. GC×GC-FID chromatogram of FOPB-03.

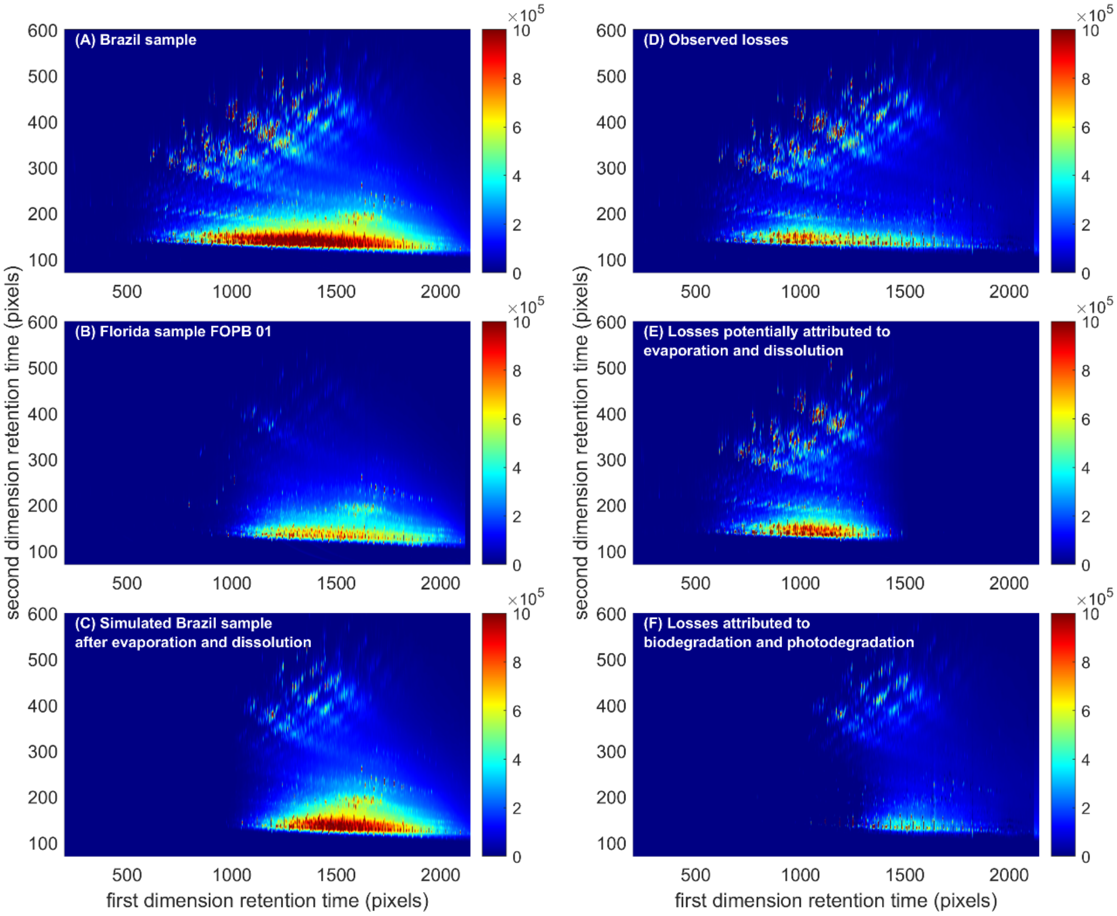

Figure S8. GC×GC-FID chromatograms of (A) Brazil-12 and (B) FOPB-01. (C) The simulated GC×GC-FID chromatogram if Brazil-12 was weathered by evaporation and aqueous dissolution after 240 days. (D) The total losses observed determined as the difference of the chromatograms (A – B). (E) The losses potentially arising from aqueous dissolution and evaporation together with unquantified contributions of biodegradation and photodegradation (A – C). (F) The losses attributed to biodegradation and photodegradation (D – E). The chromatograms were normalized according to the volume of the C_{30} hopane peak, and the FOPB-01 chromatogram was aligned to the Brazil-12 chromatogram using the algorithm of Gros et al.[11]

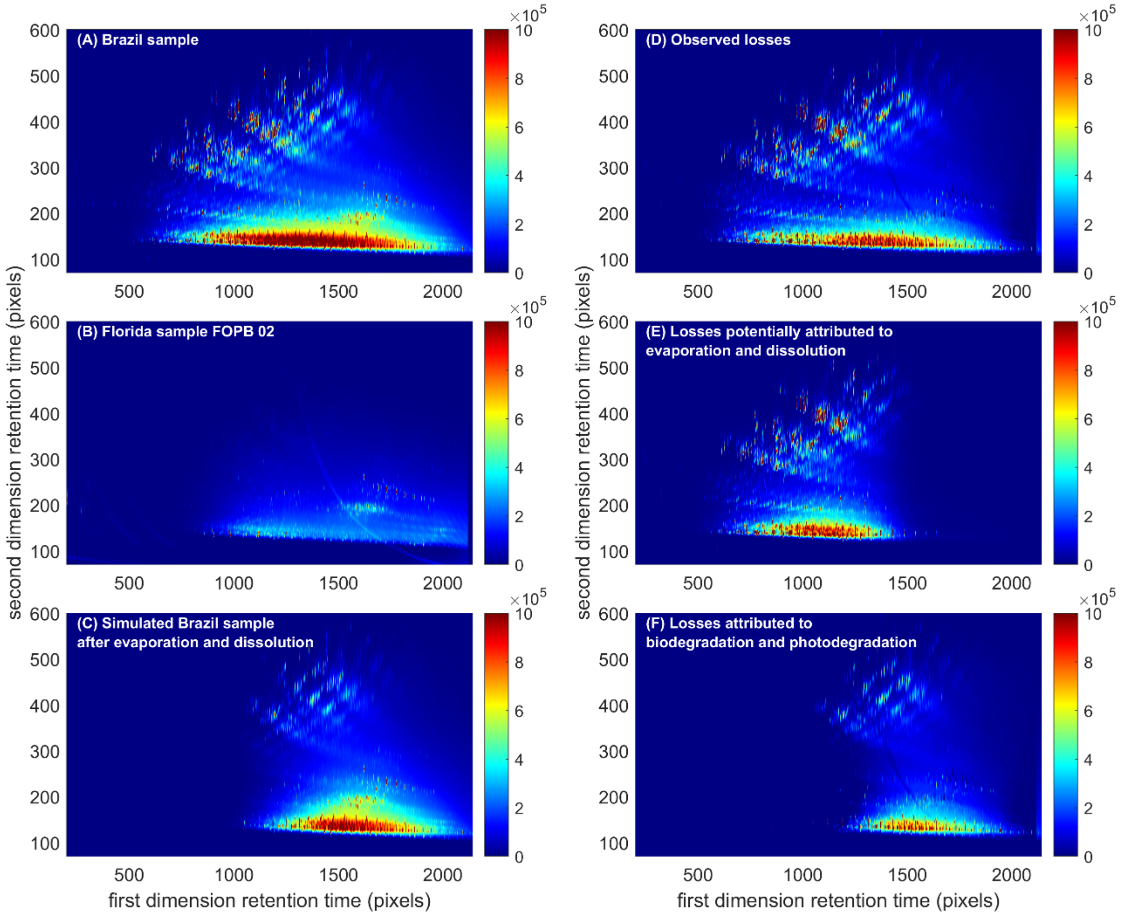

Figure S9. GC×GC-FID chromatograms of (A) Brazil-12 and (B) FOPB-02. (C) The simulated GC×GC-FID chromatogram if Brazil-12 was weathered by evaporation and aqueous dissolution after 240 days. (D) The total losses observed determined as the difference of the chromatograms (A – B). (E) The losses potentially arising from aqueous dissolution and evaporation together with unquantified contributions of biodegradation and photodegradation (A – C). (F) The losses attributed to biodegradation and photodegradation (D – E). The chromatograms were normalized according to the volume of the C_{30} hopane peak, and the FOPB-02 chromatogram was aligned to the Brazil-12 chromatogram using the algorithm of Gros et al.[11]

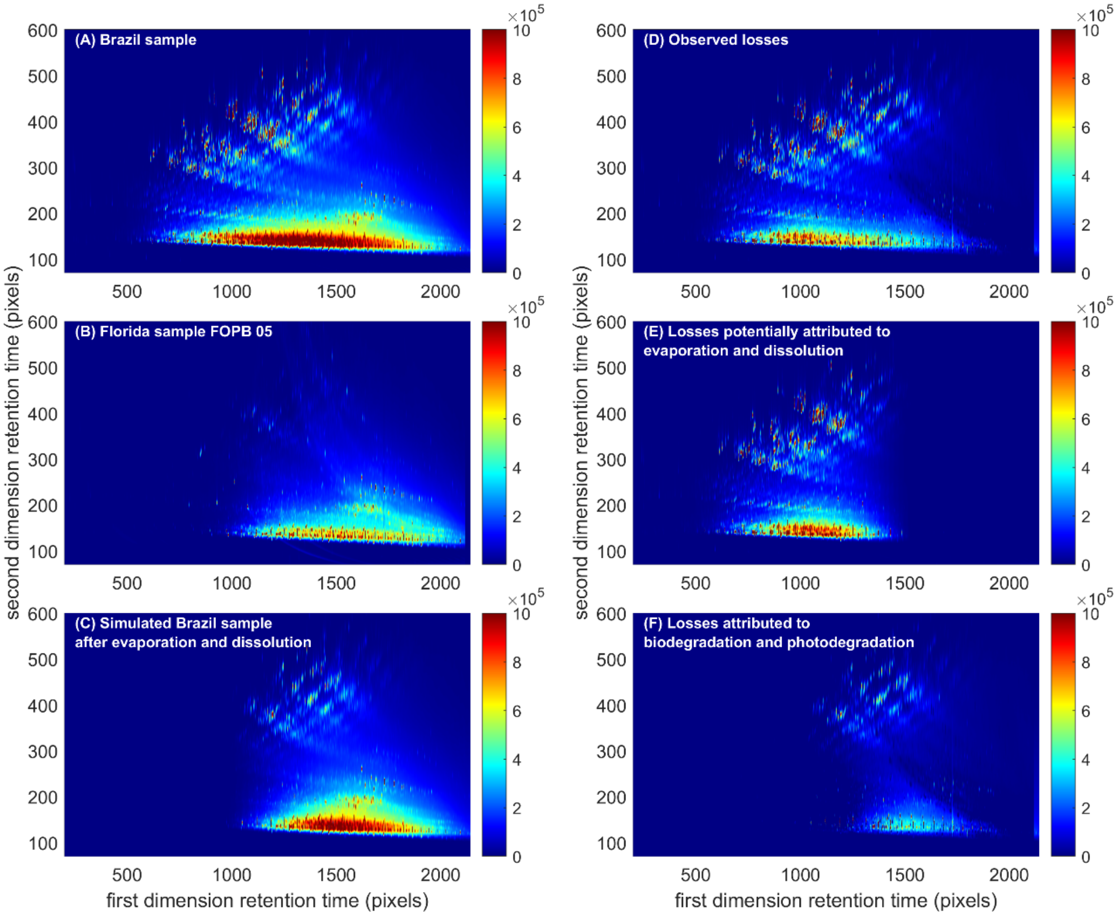

Figure S10. GC×GC-FID chromatograms of (A) Brazil-12 and (B) FOPB-05. (C) The simulated GC×GC-FID chromatogram if Brazil-12 was weathered by evaporation and aqueous dissolution after 240 days. (D) The total losses observed determined as the difference of the chromatograms (A – B). (E) The losses potentially arising from aqueous dissolution and evaporation together with unquantified contributions of biodegradation and photodegradation (A – C). (F) The losses attributed to biodegradation and photodegradation (D – E). The chromatograms were normalized according to the volume of the C_{30} hopane peak, and the FOPB-05 chromatogram was aligned to the Brazil-12 chromatogram using the algorithm of Gros et al.[11]

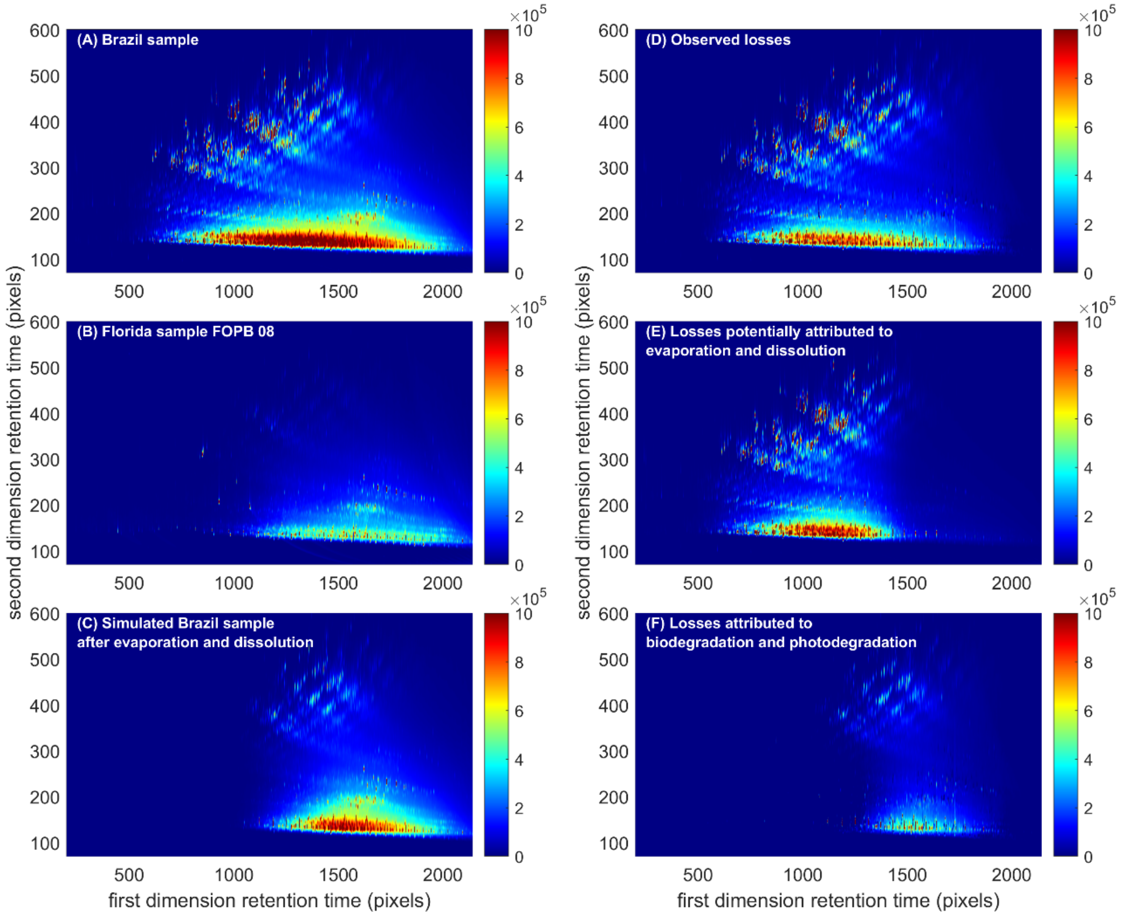

Figure S11. GC×GC-FID chromatograms of (A) Brazil-12 and (B) FOPB-08. (C) The simulated GC×GC-FID chromatogram if Brazil-12 was weathered by evaporation and aqueous dissolution after 240 days. (D) The total losses observed determined as the difference of the chromatograms (A – B). (E) The losses potentially arising from aqueous dissolution and evaporation together with unquantified contributions of biodegradation and photodegradation (A – C). (F) The losses attributed to biodegradation and photodegradation (D – E). The chromatograms were normalized according to the volume of the C_{30} hopane peak, and the FOPB-08 chromatogram was aligned to the Brazil-12 chromatogram using the algorithm of Gros et al.[11]

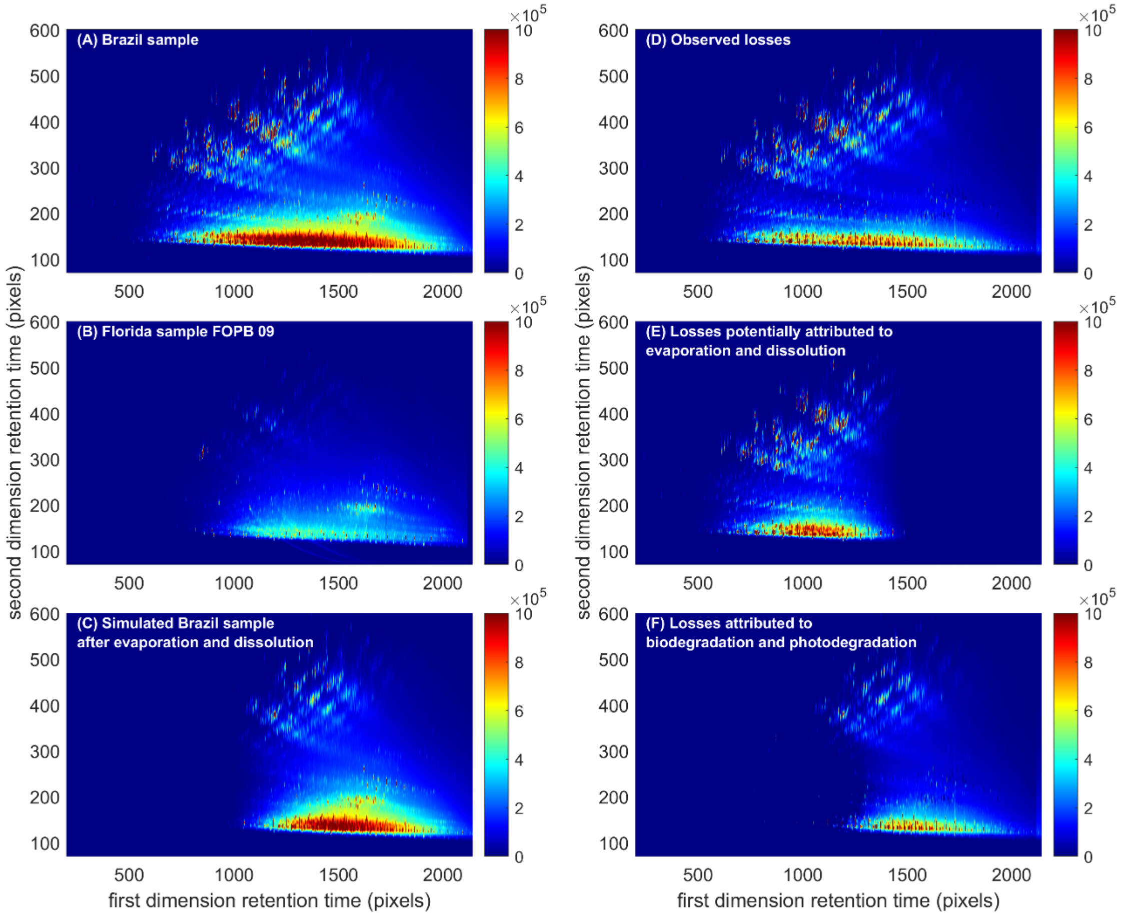

Figure S12. GC×GC-FID chromatograms of (A) Brazil-12 and (B) FOPB-09. (C) The simulated GC×GC-FID chromatogram if Brazil-12 was weathered by evaporation and aqueous dissolution after 240 days. (D) The total losses observed determined as the difference of the chromatograms (A – B). (E) The losses potentially arising from aqueous dissolution and evaporation together with unquantified contributions of biodegradation and photodegradation (A – C). (F) The losses attributed to biodegradation and photodegradation (D – E). The chromatograms were normalized according to the volume of the C_{30} hopane peak, and the FOPB-09 chromatogram was aligned to the Brazil-12 chromatogram using the algorithm of Gros et al.[11]

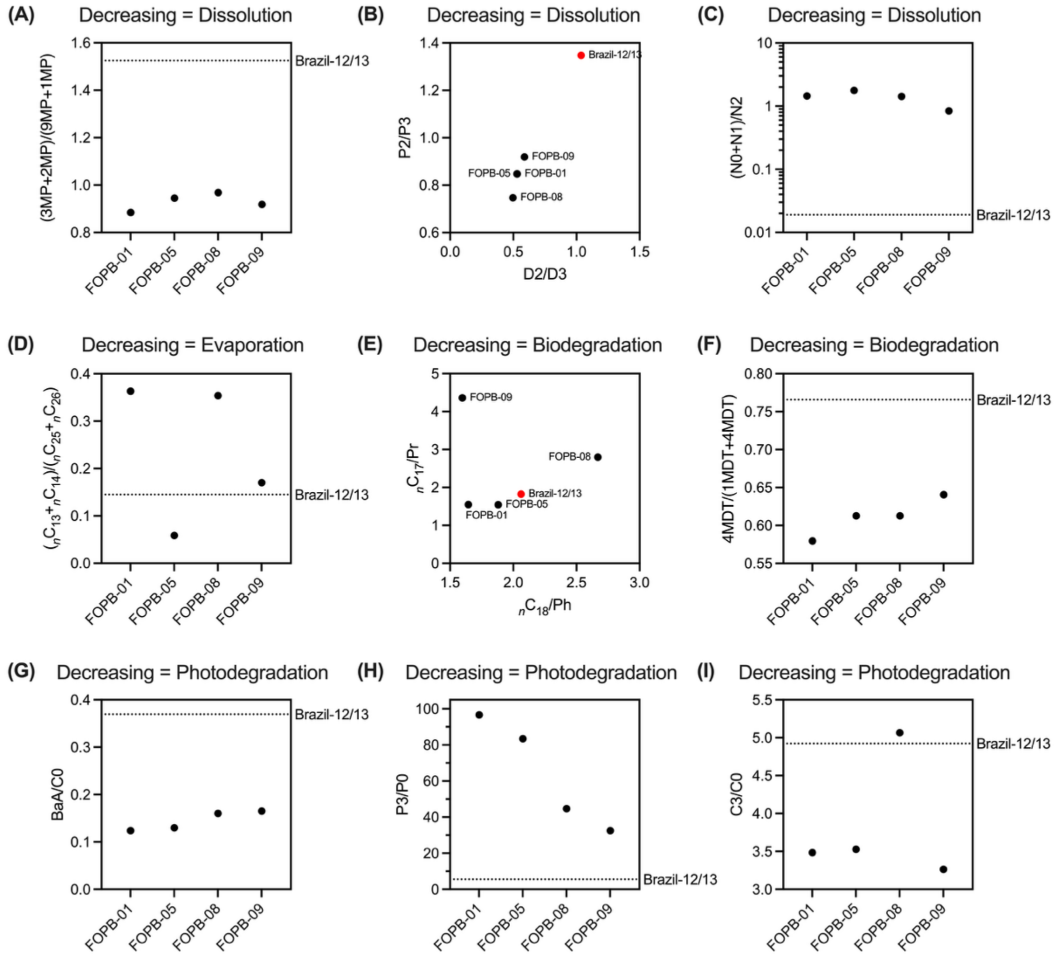

Figure S13. Diagnostic ratios of saturates and PAHs indicative of different weathering processes for the Brazil-12/13 sample and the FOPB-01, -05, -08, and -09 samples. See Supplementary Text for interpretation. Ratio of 3-methylphenathrene and 2-methylphenanthrene to 9-methylphenanthrene and 1-methylphenanthrene (A). Cross plot of the ratio of the dialkylated dibenzothiophenes to the trialkylated dibenzothiphenes and the dialkylated phenanthrenes to the trialkylated phenanthrenes (B). Ratio of naphthalene and the monoalkylated naphthalenes to the dialkylated naphthalenes (C). Ratio of *n*-tridecane and *n*-tetradecane to *n*-pentacosane and *n*-hexacosane (D). Cross plot of the ratio of *n*-heptadecane to pristane and the ratio of *n*-octadecane to phytane (E). Ratio of 4-methyldibenzothiophene to 4-methyldibenzothiophene and 1-methyldibenzothiophene (F). Ratio of benz[*a*]anthracene to chrysene (G). Ratio of trialkylated phenanthrenes to phenanthrene (H). Ratio of trialkylated chrysene to chrysene (I).

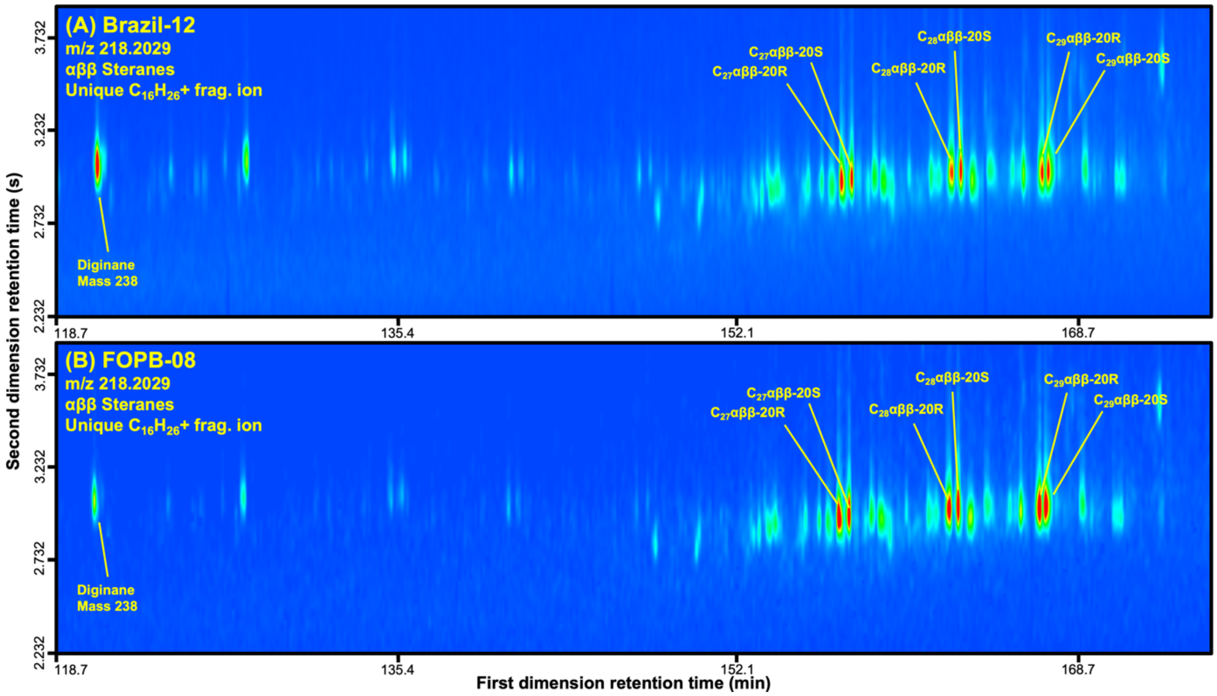

Figure S14. GC×GC-HRT extracted ion chromatograms for *m/z* 218.2029 (αββ steranes) of (A) Brazil-12 and (B) FOPB-08.

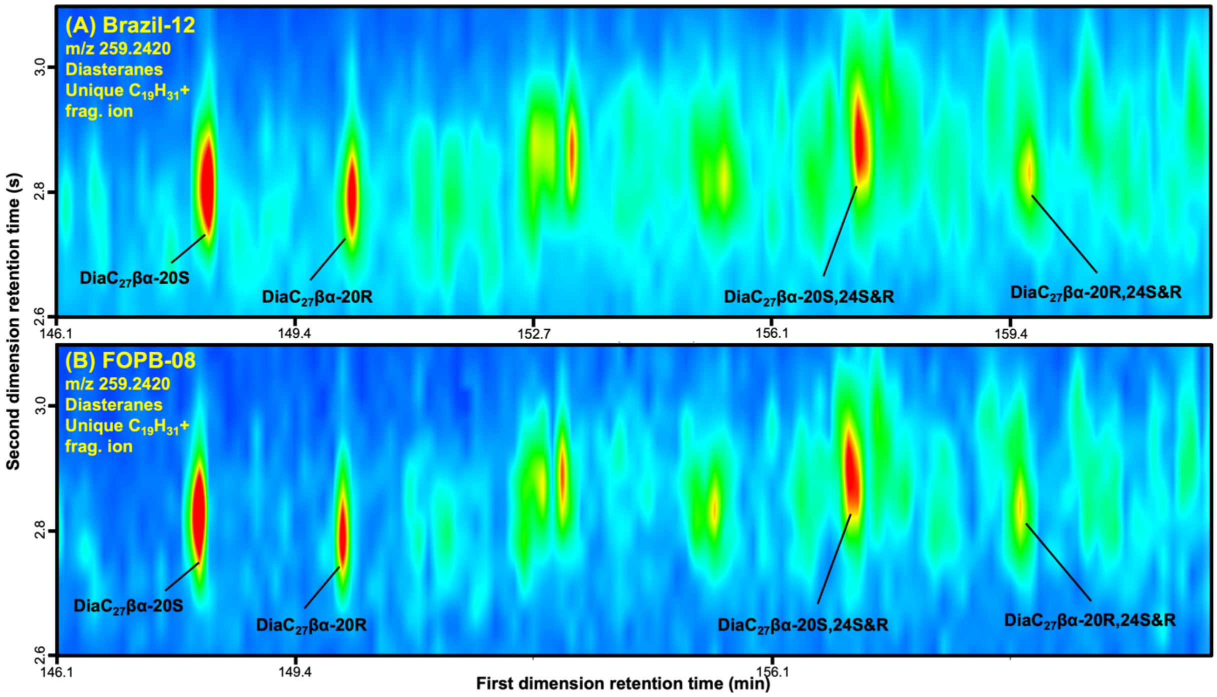

Figure S15. GC×GC-HRT extracted ion chromatograms for *m/z* 259.2420 (diasteranes) of (A) Brazil-12 and (B) FOPB-08.

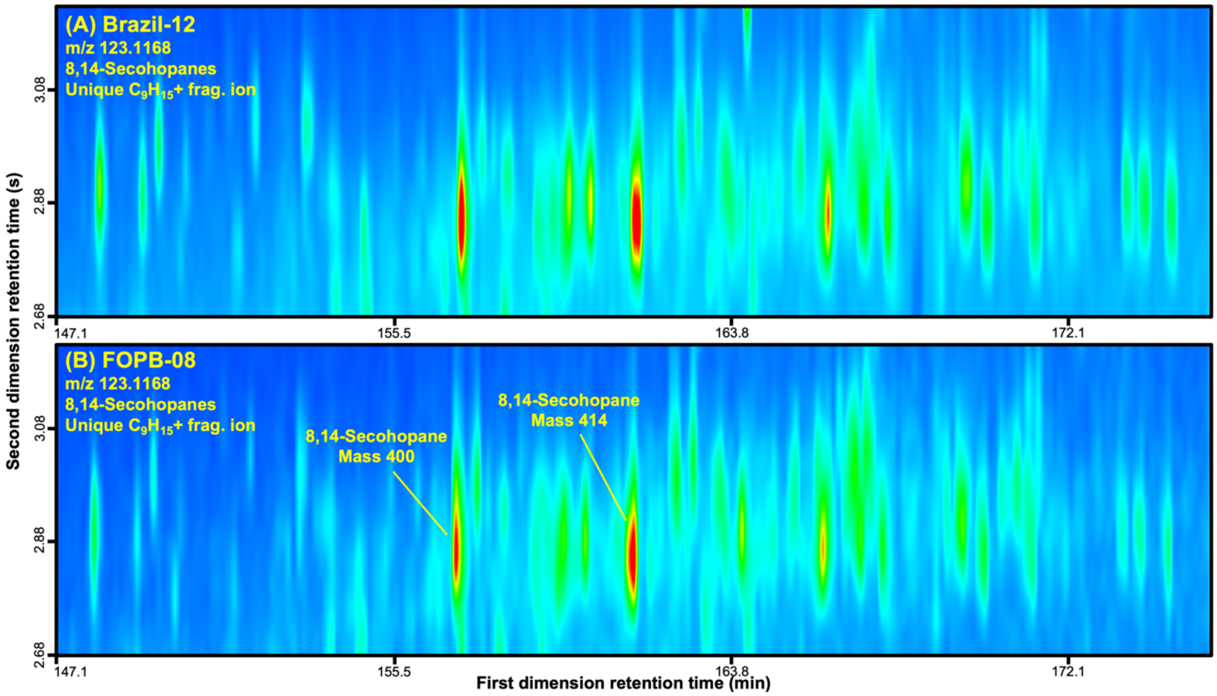

Figure S16. GC×GC-HRT extracted ion chromatograms for *m/z* 123.1168 (8,14-secohopanes) of (A) Brazil-12 and (B) FOPB-08.

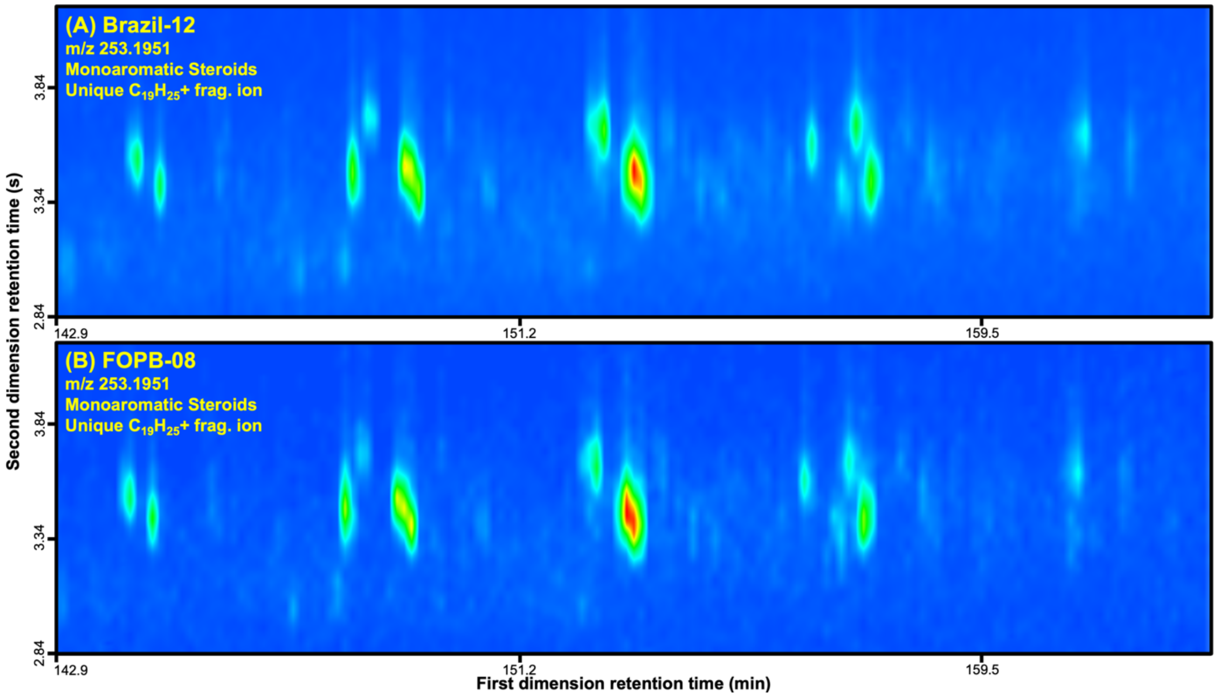

Figure S17. GC×GC-HRT extracted ion chromatograms for *m/z* 253.1951(monoaromatic steroids) of (A) Brazil-12 and (B) FOPB-08.

Table S1. FOPB samples

Sample	Collection Date	Collection Time (EST)	Location (Latitude, Longitude)
**FOPB-01**	6/23/20	8:53 AM	26º 38’ 54.4” N, 80º 02’ 16.2” W
**FOPB-02**	8/7/20	1:34 PM	26° 43' 52.5" N, 80° 02' 08.2" W
**FOPB-03**	8/7/20	1:34 PM	26° 43' 52.5" N, 80° 02' 08.2" W
FOPB-04	7/31/20	11:15 AM	26° 46' 10.2" N, 80° 02' 20.4" W
**FOPB-05**	6/4/20	10:00 AM	26º 46’ 15.8” N, 80º 02’ 17.8” W
FOPB-06	6/23/20	11:48 AM	26° 39' 17.3" N, 80° 02' 14.9" W
FOPB-07	6/11/20	1:14 PM	26° 44' 02.7" N, 80° 02' 08.8" W
**FOPB-08**	8/4/20	9:00 AM	26º 40’ 28.7” N, 80º 02’ 11.8” W
**FOPB-09**	7/27/20	12:40 PM	26° 39' 37.9" N, 80° 02' 14.8" W
FOPB-10	6/17/20	10:39 AM	26° 45' 24.3" N, 80° 02' 13.8" W

Oil was scraped for molecular analyses from several samples (in bold) (**Table S5**).

Table S2. Summary of sample analyses

Sample	GC-FID/GC-MS[1]	GC×GC-FID	GC×GC-HRT	FT-ICR-MS	Weathering simulation
FOPB-01	X	X			X
FOPB-02		X			X
FOPB-03		X			
FOPB-05	X	X			X
FOPB-08	X	X	X	X	X
FOPB-09	X	X			X
Brazil-12		X	X	X	N/A
Brazil-12/13	X				N/A

[1]Analyzed for saturated hydrocarbons, polycyclic aromatic hydrocarbons (PAHs), S-heteroaromatics, and select biomarkers by Alpha Analytical (Mansfield, MA) using United States Environmental Protection Agency (EPA) Method 8015 analysis (GC-FID; saturates) and a modified 8270D analysis (GC-MS; PAHs and biomarkers). The methods and performance details are provided elsewhere.[12]

Table S3. *n*-alkanes, select branched alkanes, and total petroleum hydrocarbons in the Brazil-12/13 and FOPB-01, -05, -08, and -09 samples (mg/kg)

Compound	Abbrev.	Brazil-12/13	FOPB-01	FOPB-05	FOPB-08	FOPB-09
Total Petroleum Hydrocarbons	TPH	247000	98800	211000	89300	162000
n-Nonane	C9	59.3	49.5	57.8	53.6	56.4
n-Decane	C10	63.8	53.2	62.1	57.6	60.6
n-Undecane	C11	59.7	49.8	58.1	53.9	56.7
n-Dodecane	C12	43.6	36.4	42.4	39.3	41.4
n-Tridecane	C13	54.9	45.8	53.4	49.5	52.1
2,6,10-Trimethyldodecane	TMD	30.1	25.1	29.3	27.1	28.6
n-Tetradecane	C14	107	25.1	29.3	27.1	28.6
2,6,10-Trimethyltridecane	TMT	107	19.9	23.2	21.5	22.6
n-Pentadecane	C15	319	19.9	23.2	21.5	22.6
n-Hexadecane	C16	509	25.1	29.3	27.2	28.6
Norpristane	NPr	197	55.1	64.3	59.6	62.7
n-Heptadecane	C17	603	55.1	64.3	108	177
Pristane	Pr	331	35.6	41.6	38.6	40.6
n-Octadecane	C18	695	50.8	126	74.2	86.4
Phytane	Ph	337	30.9	67.0	27.8	54.1
n-Nonadecane	C19	571	42.9	235	46.4	54.3
n-Eicosane	C20	848	26.7	497	35.4	107
n-Heneicosane	C21	677	33.9	582	45.5	165
n-Docosane	C22	747	32.4	673	41.3	169
n-Tricosane	C23	638	29.9	602	36.1	164
n-Tetracosane	C24	575	27.9	580	32.5	147
n-Pentacosane	C25	638	162	798	169	302
n-Hexacosane	C26	479	33.2	614	47.5	172
n-Heptacosane	C27	374	39.9	524	43.3	159
n-Octacosane	C28	358	35.8	439	38.7	131
n-Nonacosane	C29	318	111	458	120	142
n-Triacontane	C30	246	33.6	444	44.4	159
n-Hentriacontane	C31	241	28.7	474	46.2	169
n-Dotriacontane	C32	197	80.6	478	90.8	244
n-Tritriacontane	C33	211	44.2	320	45.1	128
n-Tetratriacontane	C34	132	31.0	260	56.1	118
n-Pentatriacontane	C35	149	34.7	262	64.1	140
n-Hexatriacontane	C36	104	33.2	142	35.9	77.7
n-Heptatriacontane	C37	90.4	37.1	152	40.1	84.1
n-Octatriacontane	C38	94.0	38.9	131	61.2	72.6
n-Nonatriacontane	C39	64.9	54.2	108	58.6	68.6
n-Tetracontane	C40	64.9	54.2	97.2	58.6	62.9
Total Saturated Hydrocarbons	TSH	10900	693	9060	1070	3350

Table S4. Parent and alkylated PAHs, decalins, and S-containing aromatics in the Brazil-12/13 and FOPB-01, -05, -08, and -09 samples (mg/kg)

Compound	Abbrev.	Brazil-12/13	FOPB-01	FOPB-05	FOPB-08	FOPB-09
Cis/Trans-Decalin	Dec0	0.753	0.629	0.734	0.585	0.715
C1-Decalins	Dec1	0.753	0.629	0.734	2.79	0.715
C2-Decalins	Dec2	0.753	0.629	0.734	5.31	0.715
C3-Decalins	Dec3	3.07	0.629	0.734	3.44	0.715
C4-Decalins	Dec4	16.2	0.629	0.734	0.680	0.715
Naphthalene	N0	0.862	0.720	0.840	0.778	0.819
C1-Naphthalenes	N1	1.08	0.720	0.840	1.41	0.819
C2-Naphthalenes	N2	102	0.995	0.945	1.54	1.95
C3-Naphthalenes	N3	447	0.958	1.05	1.37	2.92
C4-Naphthalenes	N4	445	1.28	2.42	2.14	5.25
2-Methylnaphthalene	2MN	0.917	0.646	0.753	1.28	0.735
1-Methylnaphthalene	1MN	0.945	0.789	0.920	0.853	0.898
Benzothiophene	BT0	0.940	0.784	0.915	0.848	0.892
C1-Benzo[*b*]thiophenes	BT1	0.940	0.784	0.915	0.848	0.892
C2-Benzo[*b*]thiophenes	BT2	10.1	0.784	1.25	0.848	1.23
C3-Benzo[*b*]thiophenes	BT3	69.6	1.11	1.86	1.30	2.12
C4-Benzo[*b*]thiophenes	BT4	96.8	0.784	1.53	1.67	2.82
Biphenyl	Bph	0.927	0.774	0.903	0.837	0.880
2,6-Dimethylnaphthalene	2,6DMN	46.4	0.595	0.694	0.644	0.677
Dibenzofuran	Dbf	1.64	0.789	0.920	0.853	0.897
Acenaphthylene	Acl	0.609	0.478	0.557	0.517	0.544
Acenaphthene	Ace	2.30	0.441	0.515	0.477	0.502
2,3,5-Trimethylnaphthalene	2,3,5TMN	49.0	0.410	0.478	0.443	0.466
Fluorene	F0	7.75	0.668	0.779	0.722	0.760
C1-Fluorenes	F1	96.7	1.02	9.16	26.0	45.7
C2-Fluorenes	F2	277	4.45	10.5	4.14	13.8
C3-Fluorenes	F3	323	22.6	47.0	14.2	43.0
Dibenzothiophene	D0	50.0	0.690	0.805	0.746	1.66
4-Methyldibenzothiophene	4MDT	118	1.97	5.33	1.66	6.27
2/3-Methyldibenzothiophene	2MDT	124	1.01	2.84	0.746	2.99
1-Methyldibenzothiophene	1MDT	36.1	1.43	3.37	1.05	3.52
C1-Dibenzothiophenes	D1	308	5.50	13.8	3.64	15.0
C2-Dibenzothiophenes	D2	598	44.3	94.7	24.1	76.5
C3-Dibenzothiophenes	D3	577	83.4	179	48.5	130
C4-Dibenzothiophenes	D4	363	58.5	130	41.5	92.1
Phenanthrene	P0	111	0.830	2.05	1.08	3.78
3-Methylphenanthrene	3MP	131	2.98	7.09	2.08	6.92
2-Methylphenanthrene	2MP	172	3.76	9.43	2.49	8.32
2-Methylanthracene	2MA	26.4	0.830	0.968	0.897	1.16
9/4-Methylphenanthrene	9MP	120	4.26	10.2	2.67	9.40
1-Methylphenanthrene	1MP	78.7	3.36	7.28	2.05	7.19
C1-Phenanthrenes/Anthracenes	P1	541	15.5	36.8	10.0	34.9
C2-Phenanthrenes/Anthracenes	P2	826	68.0	145	36.0	113
C3-Phenanthrenes/Anthracenes	P3	613	80.2	171	48.2	123
C4-Phenanthrenes/Anthracenes	P4	303	41.1	85.0	27.7	59.5
Retene	Ret	0.736	0.614	0.717	0.664	0.699
Anthracene	AN	14.1	0.516	0.602	0.558	0.822
Carbazole	Car	2.81	0.819	0.956	0.886	0.932
Fluoranthene	FL	8.00	1.17	2.24	0.860	3.36
Benzo[*b*]fluorene		13.2	0.832	1.34	0.784	1.79
Pyrene	PY	53.5	5.55	9.20	2.38	9.46
C1-Fluoranthenes/Pyrenes	FP1	187	24.1	43.4	13.6	33.9
C2-Fluoranthenes/Pyrenes	FP2	287	36.1	68.2	23.0	49.0
C3-Fluoranthenes/Pyrenes	FP3	296	43.5	86.6	30.2	62.0
C4-Fluoranthenes/Pyrenes	FP4	217	38.7	86.5	29.1	60.6
Naphthobenzothiophene	NBT0	70.9	13.4	29.9	8.60	22.3
C1-Naphthobenzothiophenes	NBT1	241	45.5	100	32.3	68.7
C2-Naphthobenzothiophenes	NBT2	384	72.4	160	53.0	107
C3-Naphthobenzothiophenes	NBT3	318	57.7	137	47.3	93.6
C4-Naphthobenzothiophenes	NBT4	213	38.9	90.7	31.5	62.2
Benz[*a*]anthracene	BaA	28.9	1.06	2.31	0.860	2.38
Chrysene/Triphenylene	C0	78.2	8.55	17.8	5.37	14.4

C1-Chrysenes	C1	262	19.9	40.4	13.9	29.1
C2-Chrysenes	C2	382	26.7	55.5	20.7	40.2
C3-Chrysenes	C3	385	29.8	62.8	27.2	47.0
C4-Chrysenes	C4	222	20.8	37.1	21.5	34.7
Benzo[*b*]fluoranthene	BbF	10.8	1.26	2.76	0.935	2.65
Benzo[*j*+*k*]fluoranthene	BjkF	2.01	NM[a]	NM[a]	NM[a]	NM[a]
Benzo[*a*]fluoranthene	BaF	0.595	0.497	0.580	0.537	0.565
Benzo[*e*]pyrene	BeP	25.2	NM[a]	NM[a]	NM[a]	NM[a]
Benzo[*a*]pyrene	BaP	16.4	1.10	1.92	0.773	1.79
Perylene	Per	7.35	0.528	1.17	0.522	0.862
Indeno[1,2,3-*cd*]pyrene	IND	2.61	NM[a]	NM[a]	NM[a]	NM[a]
Dibenz[*a*,*h*]+[*a*,*c*]anthracene	DBA	3.64	0.676	0.789	0.731	0.770
Benzo[*ghi*]perylene	Bghi	9.42	0.743	1.28	0.719	1.52

[a]NM = not measured

Table S5. Triterpanes, steranes, diasteranes, and triaromatic steroids in the Brazil-12/13 and FOPB-01, -05, -08, and -09 samples (mg/kg).

Compound	Abbrev.	Brazil-12/13	FOPB-01	FOPB-05	FOPB-08	FOPB-09
Hopane	T19	91.4	46.1	126	38.8	95.3
C23 Tricyclic Terpane	T4	90.5	21.9	47.8	16.2	34.4
C24 Tricyclic Terpane	T5	35.4	11.0	26.9	8.69	20.6
C25 Tricyclic Terpane	T6	37.4	11.4	27.9	9.99	19.8
C24 Tetracyclic Terpane	T6a	10.1	4.14	10.0	3.56	8.31
C26 Tricyclic Terpane-22S	T6b	14.9	4.23	10.8	3.55	7.74
C26 Tricyclic Terpane-22R	T6c	13.4	3.82	11.5	3.76	6.76
C28 Tricyclic Terpane-22S	T7	14.3	4.64	15.2	4.44	9.38
C28 Tricyclic Terpane-22R	T8	16.1	5.16	15.0	4.81	9.26
C29 Tricyclic Terpane-22S	T9	16.7	7.06	19.1	7.47	14.2
C29 Tricyclic Terpane-22R	T10	17.3	6.41	18.2	5.98	12.0
18a-22,29,30-Trisnorneohopane	Ts	13.9	6.25	17.5	5.46	14.6
C30 Tricyclic Terpane-22S		13.6	6.53	16.0	5.59	10.6
C30 Tricyclic Terpane-22R		15.9	4.85	15.2	4.47	9.72
17a(H)-22,29,30-Trisnorhopane	Tm	24.4	13.9	34.3	11.7	25.8
17a/b,21b/a 28,30-Bisnorhopane	T14a	17.3	9.05	24.5	7.72	17.6
17a(H),21b(H)-25-Norhopane	T14b	7.47	3.00	8.35	2.74	5.82
30-Norhopane	T15	65.0	31.5	77.8	27.5	62.9
18a(H)-30-Norneohopane	C29Ts	14.0	5.44	12.4	4.46	11.1
17a(H)-Diahopane	X	3.95	1.42	3.50	0.770	3.37
30-Normoretane	T17	9.30	3.07	9.46	2.45	5.28
18a(H)+18b(H)-Oleananes	T18	7.83	3.37	9.99	3.43	6.54
Moretane	T20	10.8	3.37	11.0	2.72	6.61
30-Homohopane-22S	T21	49.2	21.3	55.0	19.0	42.6
30-Homohopane-22R	T22	43.9	21.2	54.9	18.4	39.4
Gammacerane/C32-Diahopane		17.9	6.47	19.7	6.53	14.2
30,31-Bishomohopane-22S	T26	32.9	14.8	38.2	14.1	28.6
30,31-Bishomohopane-22R	T27	24.1	10.4	28.0	8.24	22.1
30,31-Trishomohopane-22S	T30	25.8	10.1	29.7	10.9	22.0
30,31-Trishomohopane-22R	T31	15.2	5.74	15.7	5.77	13.1
Tetrakishomohopane-22S	T32	18.7	6.86	20.7	6.87	13.0
Tetrakishomohopane-22R	T33	13.0	4.51	12.8	3.82	9.20
Pentakishomohopane-22S	T34	22.9	5.20	15.7	5.41	10.8
Pentakishomohopane-22R	T35	16.1	4.76	11.8	3.71	8.20
13b(H),17a(H)-20S-Diacholestane	S4	9.11	7.14	19.5	5.97	12.5
13b(H),17a(H)-20R-Diacholestane	S5	3.75	3.41	9.64	2.48	6.40
13b,17a-20S-Methyldiacholestane	S8	3.26	4.01	12.9	3.76	8.72
17a(H)20SC27/C29dia		15.6	13.6	37.0	11.7	25.6
17a(H)20rc27/C29dia		23.8	NM[a]	NM[a]	NM[a]	NM[a]
Unknown Sterane	S18	2.02	2.04	4.97	2.11	4.52
13a,17b-20S-Ethyldiacholestane	S19	2.62	1.66	4.01	1.14	2.84
14a,17a-20S-Methylcholestane	S20	11.0	8.30	27.9	8.43	19.5
14a,17a-20R-Methylcholestane	S24	16.2	11.7	32.8	9.34	22.9
14a(H),17a(H)-20S-Ethylcholestane	S25	19.3	16.5	45.5	11.8	31.8
14a(H),17a(H)-20R-Ethylcholestane	S28	15.3	12.1	31.7	9.88	25.7
14b(H),17b(H)-20R-Cholestane	S14	22.6	18.4	50.0	15.8	33.4
14b(H),17b(H)-20S-Cholestane	S15	21.3	17.3	47.7	16.2	32.2
14b,17b-20R-Methylcholestane	S22	22.0	18.0	51.0	15.7	37.3
14b,17b-20S-Methylcholestane	S23	27.4	22.3	61.8	18.8	42.1
14b(H),17b(H)-20R-Ethylcholestane	S26	27.0	19.6	48.9	14.4	39.1
14b(H),17b(H)-20S-Ethylcholestane	S27	15.5	16.1	47.9	17.2	35.6
C26,20R+C27,20S TAS		113	53.0	147	48.9	98.9
C28,20S TAS		57.2	35.0	106	32.8	71.6
C27,20R TAS		89.7	38.6	108	37.5	73.9
C28,20R TAS		52.9	26.9	77.1	26.8	52.9

[a]NM = not measured

Additional References

1. Brucks, J. T. Currents of the Caribbean and Adjacent Regions as Deduced from Drift-Bottle Studies. *Bull Mar Sci* 21, 455–465 (1971).
2. Luedemann, E. F. Preliminary results of drift-bottle releases and recoveries in the Western Tropical Atlantic. *Boletim do Instituto Oceanográfico* 16, 13–22 (1967).
3. Stalcup, M. C. & Metcalf, W. G. Current measurements in the passages of the Lesser Antilles. *J Geophys Res* 77, 1032–1049 (1972).
4. Richardson, P. L. Caribbean Current and eddies as observed by surface drifters. *Deep Sea Research Part II: Topical Studies in Oceanography* 52, 429–463 (2005).
5. Díez, S., Jover, E., Bayona, J. M. & Albaigés, J. *Prestige* Oil Spill. III. Fate of a Heavy Oil in the Marine Environment. *Environ Sci Technol* 41, 3075–3082 (2007).
6. Kienhuis, P. G. M., Hansen, A. B., Faksness, L.-G., Stout, S. A. & Dahlmann, G. CEN methodology for oil spill identification. in *Standard Handbook Oil Spill Environmental Forensics* (eds. Stout, S. A. & Wang, Z.) 685–728 (Elsevier, 2016). doi:10.1016/B978-0-12-803832-1.00014-3.
7. Michel, J. & Hayes, M. O. Weathering Patterns of Oil Residues Eight Years after the Exxon Valdez Oil Spill. *Mar Pollut Bull* 38, 855–863 (1999).
8. Hegazi, A. H. & Andersson, J. T. Polycyclic aromatic sulfur heterocycles as source diagnostics of petroleum pollutants in the marine environment. in *Standard Handbook Oil Spill Environmental Forensics* 313–342 (Elsevier, 2016). doi:10.1016/B978-0-12-803832-1.00006-4.
9. Yang, C. *et al.* Chromatographic Fingerprinting Analysis of Crude Oils and Petroleum Products. in *Handbook of Oil Spill Science and Technology* 93–163 (Wiley, 2014). doi:10.1002/9781118989982.ch5.
10. Lemkau, K. L. *et al.* The M/V Cosco Busan spill: Source identification and short-term fate. *Mar Pollut Bull* 60, 2123–2129 (2010).
11. Gros, J., Nabi, D., Dimitriou-Christidis, P., Rutler, R. & Arey, J. S. Robust Algorithm for Aligning Two-Dimensional Chromatograms. *Anal Chem* 84, 9033–9040 (2012).
12. Stout, S. A. Oil spill fingerprinting method for oily matrices used in the *Deepwater Horizon* NRDA. *Environ Forensics* 17, 218–243 (2016).
